# *Bifidobacterium longum* and microbiome maturation modify a nutrient intervention for stunting in Zimbabwean infants

**DOI:** 10.1016/j.ebiom.2024.105362

**Published:** 2024-09-27

**Authors:** Ethan K. Gough, Thaddeus J. Edens, Lynnea Carr, Ruairi C. Robertson, Kuda Mutasa, Robert Ntozini, Bernard Chasekwa, Hyun Min Geum, Iman Baharmand, Sandeep K. Gill, Batsirai Mutasa, Mduduzi N.N. Mbuya, Florence D. Majo, Naume Tavengwa, Freddy Francis, Joice Tome, Ceri Evans, Margaret Kosek, Andrew J. Prendergast, Amee R. Manges

**Affiliations:** a Department of International Health, Johns Hopkins Bloomberg School of Public Health; Baltimore, MD, USA; b Devil's Staircase Consulting, West Vancouver, BC, Canada; c Department of Microbiology and Immunology, University of British Columbia; Vancouver, BC, Canada; d Blizard Institute, Queen Mary University of London, London, UK; e Zvitambo Institute for Maternal and Child Health Research, Harare, Zimbabwe; f School of Population and Public Health, University of British Columbia, Vancouver, BC, Canada; g Global Alliance for Improved Nutrition, Washington, DC, 20036, USA; h Department of Experimental Medicine, University of British Columbia, Vancouver, BC, Canada; i Department of Clinical Infection, Microbiology and Immunology, University of Liverpool, Liverpool, UK; j University of Virginia School of Medicine, Charlottesville, VA, USA; k British Columbia Centre for Disease Control (BCCDC), Vancouver, BC, Canada

**Keywords:** Microbiome, Metagenome, Stunting, Nutrition, Infant

## Abstract

**Background:**

Small-quantity lipid-based nutrient supplements (SQ-LNS), which has been widely tested to reduce child stunting, has largely modest effects to date, but the mechanisms underlying these modest effects are unclear. Child stunting is a longstanding indicator of chronic undernutrition and it remains a prevalent public health problem. The infant gut microbiome may be a key contributor to stunting; and mother and infant fucosyltransferase (FUT) phenotypes are important determinants of infant microbiome composition.

**Methods:**

We investigated whether mother-infant FUT status (n = 792) and infant gut microbiome composition (n = 354 fecal specimens from 172 infants) modified the impact of an infant and young child feeding (IYCF) intervention, that included SQ-LNS, on stunting at age 18 months in secondary analysis of a randomized trial in rural Zimbabwe.

**Findings:**

We found that the impact of the IYCF intervention on stunting was modified by: (i) mother-infant FUT2+/FUT3− phenotype (difference-in-differences −32.6% [95% CI: −55.3%, −9.9%]); (ii) changes in species composition that reflected microbiome maturation (difference-in-differences −68.1% [95% CI: −99.0%, −28.5%); and (iii) greater relative abundance of *B. longum* (differences-in-differences 49.1% [95% CI: 26.6%, 73.6%]). The dominant strains of *B. longum* when the intervention started were most similar to the proficient milk oligosaccharide utilizer subspecies *infantis*, which decreased with infant age and differed by mother-infant FUT2+/FUT3− phenotypes.

**Interpretation:**

These findings indicate that a persistently “younger” microbiome at initiation of the intervention reduced its benefits on stunting in areas with a high prevalence of growth restriction.

**Funding:**

10.13039/100000865Bill and Melinda Gates Foundation, UK DFID/Aid, 10.13039/100010269Wellcome Trust, 10.13039/100009131Swiss Agency for Development and Cooperation, US 10.13039/100000002National Institutes of Health, 10.13039/100006641UNICEF, and 10.13039/501100001720Nutricia Research Foundation.

**Research in context**

**Evidence before this study**

Chronic child undernutrition, as indicated by stunted linear growth, remains an important global health challenge in low- and middle-income countries that poses life-long economic and health-related consequences. Small-quantity lipid-based nutrient supplements (SQ-LNS) to improve infant and young child feeding (IYCF) have been tested in a variety of contexts to improve chronic child undernutrition, but effects on stunting have been modest. Reasons for the modest impact of interventions that include SQ-LNS are unclear, but are important to understand in order to develop more effective strategies. The gut microbiome plays an important role in infant health and development; and in infancy, human milk oligosaccharide composition (HMO) and infant gut glycan expression are key determinants of gut microbiome composition. Animal models suggest that infant gut microbiome composition modifies the effect of diet and nutrient supplements on undernutrition phenotypes. However, studies that investigate the effect of the gut microbiome on SQ-LNS efficacy in human infants are lacking, and the determinants of microbiome features that can modify the efficacy of SQ-LNS are unknown.

**Added value of this study**

We used shotgun metagenomics performed on infant fecal specimens collected longitudinally from 1 to 18 months of age to investigate whether mother and infant fucosyltransferase phenotypes, which determine HMO composition and infant gut glycan expression, and infant gut microbiome composition modified the impact of a IYCF intervention, that included SQ-LNS, on stunting at age 18 months in the Sanitation, Hygiene, Infant Nutrition Efficacy trial in rural Zimbabwe. We found that the impact of the IYCF intervention on stunting was reduced by paired fucosyltransferase 2 and 3 phenotypes between mothers and their infants, delayed microbiome age, and greater *Bifidobacterium longum* relative abundance when the IYCF intervention started. The predominant *B. longum* strains at that time were most similar to subspecies *infantis*, which is highly specialized to breast milk and utilization of HMOs. Paired fucosyltransferase 2 and 3 phenotypes between mothers and their infants predicted both *B. longum* relative abundance and detection of subspecies *infantis* strains over time. This is the first study to identify features of the gut microbiome that modify the effect of an intervention that includes SQ-LNS, to improve IYCF, on chronic undernutrition in infants, and to identify biological determinants of those features.

**Implications of all the available evidence**

Lower microbiota maturation is a characteristic of severe acute malnutrition. Nutrient supplements, such as ready-to-use therapeutic food, only transiently improve microbiome age. Other nutrient supplement formulations that are designed to improve microbiome age by promoting the growth of weaning-phase taxa, termed microbiota-directed complementary food (MDCF), have a modest impact on acute undernutrition in infants and children in the short-term, but may require greater duration of intervention to bolster these effects, and have no impact on chronic undernutrition to date. Furthermore, long-term maturation of the infant microbiome by MDCF has not yet been reported. Interventions that include existing SQ-LNS formulations can be made more effective at reducing chronic undernutrition if started when the infant gut microbiome is adequately mature. Thus, pairing interventions to promote optimal maturation of the infant gut microbiome with SQ-LNS at the time when complementary feeding starts may be a more effective strategy to reduce infant stunting in LMICs. Elucidating the biological drivers of appropriate maturation, such as variations in maternal HMOs and infant gut glycans, will inform development of such next generation interventions that harness HMOs and infant gut glycans to boost the efficacy of interventions that include SQ-LNS on stunting.

## Introduction

Globally, 21% of children under 5 years of age (149 million) are stunted,^1^ defined as deficits in linear growth.^2^ Stunting is an indicator of chronic undernutrition that largely accrues from conception to 24 months of age,^3^^,^^4^ and is associated with reductions in child survival, neurodevelopment, educational attainment, and adult economic productivity.^5^ Small-quantity lipid-based nutrient supplements (SQ-LNS) is a broadly tested intervention to improve infant and young child feeding (IYCF) to reduce chronic undernutrition, starting at 6-months (mo) of age, which is the recommended time for introduction of complementary foods. Randomized controlled trials (RCT) of interventions that involve SQ-LNS provision to infants have shown small reductions in stunting (12% relative reduction), but effects have varied.^6^ Evidence to support other nutrition interventions is also limited.^7^ Elucidating the reasons for the limited impact is crucial to the development of more effective strategies.

The human microbiome has been shown to impact infant health,^8^ and studies suggest a role of the intestinal microbiome in child nutritional status, particularly acute undernutrition as indicated by ponderal (weight) growth.^9^ In early infancy, the gut microbiome is influenced primarily by breastfeeding, with differences persisting beyond age 6 mo.^10^ Breastfeeding-associated differences are partly driven by differences in human milk oligosaccharide (HMO) composition, which are metabolized by specific commensal bacteria, in particular, Bifidobacterium, and thereby influence bacterial growth and activity.^11^^,^^12^ *Bifidobacterium longum* subspecies *infantis*, for example, is adapted to utilize a large variety of HMOs.^11^^,^^12^ HMOs, in combination with commensal gut bacteria, have also been shown to improve ponderal growth in animal models of acute undernutrition.^13^

Active maternal α-1,2-fucosyltransferase (FUT2) and α-1,3-fucosyltransferase (FUT3) genes are key determinants of HMO composition^14^, ^15^, ^16^ and of host glycan production more broadly.^17^^,^^18^ Individuals with at least one functional FUT2 or FUT3 allele produce fucosylated histo-blood group antigens (HBGA) and HMOs; by contrast, individuals lacking two functional alleles do not.^18^ FUT2 and FUT3 phenotypes, thereby, drive inter-individual HBGA and HMO diversity (Figure S1).^17^^,^^19^ As such, in addition to the impact of maternal FUT2/FUT3 status on the infant gut microbiome via their influence on HMO composition, host FUT2/FUT3 phenotypes also determine tissue surface HBGA expression in the gut, which may also affect the microbiome through the availability of host glycans, fucose, and sialic acid, as sources of carbon for gut bacteria, and through competition for adhesion sites.^20^, ^21^, ^22^

There is growing interest in the moderating effect of the gut microbiome on nutritional interventions. The impact of dietary interventions for weight reduction is modified up to 4-fold by microbiota composition in RCTs.^23^^,^^24^ In observational studies, microbiome composition also modified the association between diet and biomarkers of metabolic syndrome by up to 2-fold.^25^ In addition, a synergistic effect of microbiome composition and diet on undernutrition phenotypes was demonstrated using animal models.^26^^,^^27^

Here we aimed to determine the effect of the infant gut microbiome on efficacy of an IYCF intervention that included SQ-LNS to improve indicators of chronic undernutrition at age 18 mo, specifically stunting (primary outcome) and length-for-age z-score (LAZ) (secondary outcome). We hypothesized that the efficacy of IYCF, when started at age 6 mo, on these outcomes is modified by mother and infant FUT2/FUT3 phenotype, as drivers of variation in gut microbiome composition, and by the infant gut microbiome. We tested this hypothesis using data from HIV-unexposed infants enrolled in the Sanitation Hygiene Infant Nutrition Efficacy (SHINE) trial conducted in rural Zimbabwe, in which the IYCF intervention reduced stunting by 20% and increased LAZ by 0.16 standard deviations at age 18 mo.^28^

## Methods

### Ethics

All SHINE mothers provided written informed consent. The Medical Research Council of Zimbabwe (MRCZ/A/1675), Johns Hopkins Bloomberg School of Public Health (JHU IRB #4205), and the University of British Columbia Ethics Board (H15-03074) approved the study protocol, including the microbiome analyses.

### Study design

The SHINE trial (NCT01824940) was a 2 × 2 factorial cluster-randomized trial that enrolled 5280 pregnant women to test the impact of improved household water quality, sanitation, and hygiene (WASH) and improved infant and young child feeding (IYCF) via provision of SQ-LNS to the infant from age 6 mo to 18 mo, on linear growth and anemia at age 18 mo in rural Zimbabwe. A detailed description of the SHINE trial design and methods has been published.^29^ A subset of mother-infant pairs was invited to join a substudy to collect additional biological specimens at 1, 3, 6, 12 and 18 months of infant age. The analyses presented here utilize data and specimens from HIV-uninfected mothers and their infants enrolled in the specimen collection substudy.

The fecal microbiome was characterized in 354 specimens collected from 172 HIV-unexposed infants from 1 to 18 mo of age. Specimen selection criteria have been previously reported^30^ (Supplementary methods). Species composition was determined using MetaPhlAn3.0^31^ and details have been previously reported.^30^ UniProt gene family profiles were generated for dominant *B. longum* strains in 218 SHINE metagenomes, from 136 infants, with sufficient coverage of *B. longum* for pangenome analysis (Figure S2), and for 118 *B. longum* reference strains using PanPhlan3.0.^31^ To facilitate interpretation of UniProt gene family profiles, biological pathways were identified in each strain using MinPath with default settings^32^ and the MetaCyc database.^33^

### Assessment of FUT2 and FUT3 status

Saliva samples were collected by oral swab from mothers and infants to assess FUT2/FUT3 status using a previously reported phenotyping assay.^34^ To more precisely reflect the potential for joint activity of these enzymes in HBGA and HMO production (Figure S1), we defined FUT2/FUT3 phenotype combinations as FUT2+/FUT3+ (Lewis-positive secretors), FUT2+/FUT3− (Lewis-null secretors), or FUT2−/FUT3+ (Lewis-positive non-secretors) (Table S2). In order to determine the impact of concordance in maternal and infant HBGA on efficacy of the IYCF intervention, we further defined paired mother-infant phenotypes using the combinations of maternal and infant FUT2/FUT3 phenotypes presented in Table S3. Each paired mother-infant phenotype was classified as *both* (if mother and infant shared the same phenotype), *none* (if neither had the phenotype), *infant only* (if the infant had the phenotype but the mother did not) or *mother only* (if the infant did not have the phenotype but the mother did).

### Statistics

#### β-diversity analyses

To explore sources of variation in microbiome composition and derive interpretable measures of microbiome species turnover, we identified infant characteristics that explain species β-diversity using constrained principal coordinates analysis (PCoA) of Bray–Curtis dissimilarities (*capscale*)^35^ between 348 metagenomes from 170 infants with available data on characteristics of interest. Characteristics of interest included factors that are known to be correlated with microbiome composition (infant age, sex, EBF at 3mo, minimum dietary diversity, maternal and infant FUT2/FUT3 phenotype). Statistical significance was tested by PERMANOVA of distance matrices with 1000 permutations (*adonis2*).^36^ We then developed a final multivariable constrained PCoA model that included covariates which explained a significant fraction of the variance in microbiome composition (p < 0.05), as well as mother-infant FUT2/FUT3 phenotypes given our *a priori* hypothesis that mother and infant fucosyltransferase phenotypes modify efficacy of the IYCF intervention by impacting infant microbiome composition. We derived four PCoA axis scores from this final multivariable model which represented changes in microbiome composition (species turnover) along gradients defined by each infant characteristic included in the full model.

#### Biological interaction analyses

First, we assessed interaction between randomization to the IYCF intervention and mother-infant FUT2/FUT3 phenotype on the difference scale using multivariable regression. The difference scale is appropriate for statistical estimation of synergistic biological effects,^37^ and controlling the false discovery rate.^38^ The primary outcome was stunting at 18mo. We used generalized linear models (*glm*) with a Gaussian distribution, an identity link, and sandwich standard errors (*sandwich*)^39^ of the following form

$$P\left[{Stunted}_{18mo\;visit}\right]=\beta_{0}+\beta_{1}\times {FUT2/FUT3}_{none}+\beta_{2}\times {FUT2/FUT3}_{only\;infant}+\beta_{3}\times {FUT2/FUT3}_{only\;mother}+\beta_{4}\times IYCF+\beta_{5}\times \left(IYCF\times {FUT2/FUT3}_{none}\right)+\beta_{6}\times \left(IYCF\times {FUT2/FUT3}_{infant\;only}\right)+\beta_{7}\times \left(IYCF\times {FUT2/FUT3}_{mother\;only}\right)+\beta_{8}\times \mathrm{Infant}\;Sex+\beta_{9}\times {Infant\;Age}_{6mo\;visit}+\beta_{10}\times {LAZ}_{6mo\;visit}+\beta_{11}\times infant\;met\;minimum\;dietary\;diversity$$

Models included terms for the interaction between IYCF and paired mother-infant FUT2/FUT3 status, using dummy variables for the previously described *none*, *only infant*, and *only mother* phenotypes with the *both* phenotype as the referent. Models also included allocation to the IYCF intervention, infant sex, paired mother-infant FUT2/FUT3 status, infant age at specimen collection, LAZ at specimen collection, and an indicator of whether infants met minimum dietary diversity. We did not include WASH arm because, in prior analyses, the SHINE WASH intervention did not affect stunting or LAZ at 18 mo^28^ nor infant gut microbiome composition.^30^ Since the IYCF intervention was started at the 6 mo follow-up visit, our interaction models used covariate data from that visit. Models included 610 infants with FUT2/FUT3 assessed in both infants and their mother and available covariate data (Figure S2). We fitted a separate model for each paired mother-infant FUT2/FUT3 phenotype (FUT2+/FUT3+, FUT2+/FUT3−, or FUT2−FUT3+). Interaction term coefficients are directly interpreted as the difference (increase or decrease) in the effect of the IYCF intervention on stunting at 18 mo when comparing each FUT2/FUT3 phenotype subgroup to the referent subgroup.

Next, we assessed modification of the IYCF intervention by microbiome composition using multivariable regression, as in the previous section, with a term for the interaction between allocation to the IYCF intervention and PCoA axis scores derived from our final multivariable constrained PCoA model. Models were restricted to 53 infants who had a fecal specimen collected at the 6 mo follow-up visit when the IYCF intervention started (Figure S2). Likewise, while our final constrained PCoA model used all sequenced specimens collected during follow-up, we used the derived PCoA axis scores corresponding to the 6 mo visit in our interaction models. Models included covariates as described above. Models were of the following form

$$P\left[{Stunted}_{18mo\;visit}\right]=\beta_{0}+\beta_{1}\times PCoA\;score+\beta_{2}\times IYCF+\beta_{3}\times \left(IYCF\times PCoA\;score\right)+\beta_{4}\times Infant\;Sex+\beta_{5}\times {Infant\;Age}_{6mo\;visit}+\beta_{6}\times {LAZ}_{6mo\;visit}+\beta_{6}\times infant\;met\;minimum\;dietary\;diversity$$

PCoA axis scores were modelled as continuous variables to avoid estimating interaction effects within sparse subgroups in this smaller subset of infants. We fitted a separate interaction model for each PCoA axis score. p-values were adjusted for multiple hypothesis testing to preserve the false discovery rate (FDR).^40^ Interaction term coefficients are directly interpreted as the change (increase or decrease) in the effect of the IYCF intervention on stunting at 18mo per one unit increase in PCoA axis score.

To determine if differences in the relative abundances of specific microbiome species that characterized the constrained PCoA axes were also associated with significant effect modification of the IYCF intervention, we fitted multivariable regression models that included IYCF-by-species relative abundance interaction terms and prespecified covariates as described in the previous paragraph. Models were also restricted to 53 infants who had a fecal specimen collected at the 6mo follow-up visit when the intervention started (Figure S2). We winsorized the relative abundance of *B. pseudocatenulatum* at the 90th percentile to minimize the effect of influential outliers on estimation of regression coefficients.^41^ Relative abundances were centered and scaled to standard deviation units, and were modelled as continuous variables to avoid estimating interaction effects within sparse subgroups in this smaller subset of infants. We fitted a separate model for each microbiome species of interest, defined as those strongly associated with PCoA axis 1 to 4 scores (loadings >0.5 or < −0.5) (Figures S4–S7). Interaction term coefficients are directly interpreted as the change (increase or decrease) in the effect of the IYCF intervention on stunting at 18 mo per one standard deviation increase in species relative abundance.

Analyses were repeated with LAZ at the 18 mo visit (secondary outcome) as the dependent variable.

#### Identification and analysis of *Bifidobacterium longum* strain clusters

*B. longum* strain profiles produced with PanPhlan3.0, which indicate whether UniProt gene families are present in a strain,^31^ were converted to Jaccard dissimilarity matrices and visualized by PCoA to ascertain the existence of strain clusters (*capscale*).^35^ Three clusters were identified. To determine strain cluster membership, we performed hierarchical clustering of Jaccard dissimilarities with Ward's error sum of squares algorithm (*hclust*)^42^ and cut the cluster dendrogram at a height to obtain three clusters. These analyses were applied to 218 SHINE and 118 reference *B. longum* pangenomes.

Differences in UniProt gene family profiles between *B. longum* strain clusters were determined by two-sided Fisher's Exact test (*fisher.test*), with adjustment for multiple hypothesis testing to preserve the FDR,^40^ and were visualized using heatmaps (*heatmap3*). 3260 UniProt gene families were differentially present between strain clusters after FDR-adjustment. We performed overrepresentation analyses^43^ using one-sided Fisher's Exact tests to determine whether the differentially present gene families were more likely to function as particular CAZymes,^44^ transporters,^45^ or in specific GO biological processes.^46^ These analyses were repeated using the biological pathways determined with MinPath.^32^ 115 pathways were differentially present between strain clusters after FDR-adjustment. We performed overrepresentation analyses of these pathways to determine whether they were more likely to have particular biological functions defined by MetaCyc pathway types.^33^ These analyses were applied to 218 SHINE *B. longum* pangenomes to make inferences about differences in metabolic potential between SHINE strains (Figure S2).

#### Predictors of Bifidobacterium relative abundance and strain detection

Predictors of longitudinal *B. longum* relative abundance were assessed using mixed-effects zero-inflated beta regression estimated by restricted maximum likelihood (*gamlss*).^47^ The model included infant age at specimen collection, sex, exclusive breastfeeding (EBF) status at 3mo of infant age, an indicator of whether infants’ diet met minimum dietary diversity, and mother-infant FUT2/FUT3 phenotypes, with random intercepts (*re*) and a first order autocorrelation structure (*corCAR1*). Models included 225 specimens from 87 infants who had more than one specimen collected and covariate data (Figure S2).

Predictors of longitudinal *B. longum* strain cluster were assessed by logistic regression (*glm*) using an indicator of strain cluster presence as the dependent variable, with the same covariates, and sandwich standard errors. We fitted a separate model for each cluster. Bias corrected^48^ logistic regression was used to facilitate stable parameter estimation due to separation resulting from the small sample size.^49^ Models included 133 specimens from 70 infants who had more than one specimen collected and pangenome strain cluster data (Figure S2).

All statistical analyses were conducted in R version 4.2.0.

### Role of funders

The funders of this study had no role in data collection, analysis, or interpretation, trial design, patient recruitment, writing of this report or any aspect pertinent to the study.

## Results

### Mother-infant FUT2+/FUT3− phenotype discordance modifies the effect of IYCF on stunting at 18 mo

Overall FUT2 and FUT3 phenotype frequencies among mothers and infants are described in Table S2 and the Supplementary methods. To determine the impact of maternal and infant HBGA phenotypes on efficacy of the IYCF intervention, we investigated whether concordance in FUT2/FUT3 phenotypes between pairs of infants and their mothers modified the effect of the IYCF intervention on stunting or LAZ at 18 mo using multivariable regression. We defined these paired mother-infant phenotypes as *both* (if mother and infant shared the same phenotype), *none* (if neither had the phenotype), *infant only* (if the infant had the phenotype but the mother did not) or *mother only* (if the infant did not have the phenotype but the mother did) (Table S3). Amongst infants who were randomized to the IYCF intervention, those who were in the *mother only* FUT2+/FUT3− group (13.5%) had a 33.6% (95% CI: −55.3%, −9.9%) decreased prevalence of stunting than infants in the *both* FUT2+/FUT3− group (11.1%) (Table 1). The *infant only* (11.1%) and *none* (64.3%) FUT2+/FUT3− groups, and other mother-infant FUT2/FUT3 phenotype combinations, did not show evidence of effect modification (Table S5), and there was no evidence of effect modification on LAZ after adjustment for multiple testing (Table S6). Overall, our results indicate that discordance between mothers and infants in FUT2+/FUT3− phenotype, whereby mothers had the phenotype and infants did not, was associated with increased efficacy of IYCF to reduce stunting.

**Table 1:** Multivariable regression model^a^ to estimate modification of IYCF on stunting at 18 mo by mother-infant FUT2+/FUT3− phenotype among 610 infants in whom mother and infant FUT2 and FUT3 status was ascertained.

	Main effects			IYCF-by-FUT2 and FUT3 phenotype combination interaction effects		
	PD (95% CI)	p-value	Adjusted p-value^b^	Prevalence difference-in-differences (95% CI)	p-value	Adjusted p-value^b^
Both FUT2+/FUT3−	ref	ref	ref	ref	ref	ref
None FUT2+/FUT3−	12.9% (0.6%, 25.2%)	0.041	0.122	−21.5% (−41.9%, −1.0%)	0.039	0.118
Infant only FUT2+/FUT3−	15.2% (−2.6%, 33.0%)	0.094	0.283	−21.1% (−48.2%, 6.0%)	0.127	0.191
Mom only FUT2+/FUT3−	28.9% (13.6%, 44.3%)	<0.001	0.001	−32.6% (−55.3%, −9.9%)	0.005	0.015
IYCF	16.5% (−1.8%, 34.8%)	0.077	0.177			

PD, prevalence difference (in percentage points); 95% CI, 95% confidence interval; IYCF, infant and young child feeding.

a Covariates include birthweight, infant sex, age at the 6 mo visit, infant LAZ at the 6 mo visit, infant diet diversity score at the 6 mo visit, and mother-infant Lewis-null secretor phenotype discordance coded as both, none, infant only or mother only. N's are reported in Table S4.

b Adjusted for multiple hypothesis testing by the Benjamini-Hochberg method.

### Infant gut microbiome composition modifies the effect of IYCF on stunting at 18 mo

Given our finding that infants whose mothers were FUT2+/FUT3−, while the infant was not, had greater reductions in stunting prevalence by the IYCF intervention; and considering the potential importance of FUT2/FUT3 phenotypes on early infant microbiome composition via variation in maternal HMO composition and infant gut HBGA expression, we next investigated whether microbiome species composition showed evidence for modification of the IYCF intervention. Species composition was quantified using the four PCoA axis scores derived from our final constrained multivariable PCoA model, which represented variation in microbiome composition due to infant age and *none*, *infant only*, or *mother only* FUT2+/FUT3− phenotype compared to *both*. PCoA axis 1 and 2 scores showed evidence of interaction with the IYCF intervention on stunting at 18 mo (Table 2), but not LAZ (Table S8). As described in more detail in the next section, greater axis 1 scores represented species turnover with increasing infant age (Figure S3), thus reflecting microbiome maturation; while greater axis 2 scores represented differences in species composition associated with mother-infant FUT2+/FUT3− phenotypes (Figure S3). Amongst infants randomized to the IYCF intervention, each one unit increase in PCoA axis 1 scores at age 6mo was associated with 68.1% (95% CI: −99.0%, −28.5%, adjusted p = 0.003) decreased prevalence of stunting at 18 mo (Table 2). In contrast, amongst infants randomized to the IYCF intervention, each one unit increase in PCoA axis 2 scores was associated with 13.3% (95% CI: 6.4%, 20.3%, adjusted p = 0.001) increased prevalence of stunting at 18 mo (Table 2). Taken together, our findings showed that infant age-related microbiome species maturation was associated with greater reduction in stunting at 18mo by the IYCF intervention; while microbiome species composition related to mother-infant FUT2+/FUT3− phenotype was associated with lesser reduction in stunting, suggesting variation in microbiome composition due to these infant characteristics is an important determinant of the intervention's efficacy to reduce stunting.

**Table 2:** Multivariable regression models^a^ to estimate modification of IYCF on stunting at 18 mo by infant gut microbiome species turnover in 53 infants in whom fecal specimens were available at the 6-month follow-up visit and stunting status was ascertained at the 18-month follow-up visit.

	n/N^b^	PD (95% CI)	p-value	Adjusted p-value^c^
PCoA Axis 1				
IYCF	17/53	−49.7% (−99.0%, −14.2%)	0.006	0.029
PC1		22.8% (−99.0%, 46.6%)	0.061	0.158
PC1-by-IYCF^d^		−68.1% (−99.0%, −28.5%)	0.001	0.003
PCoA Axis 2				
IYCF	17/53	−19.2% (−99.0%, 4.4%)	0.111	0.242
PC2		−5.9% (−99.0%, −0.7%)	0.026	0.089
PC2-by-IYCF^d^		13.3% (6.4%, 20.3%)	<0.001	0.001
PCoA Axis 3				
IYCF	17/53	2.1% (−99.0%, 30.1%)	0.882	0.921
PC3		3.2% (−99.0%, 8.5%)	0.224	0.316
PC3-by-IYCF^d^		−8.7% (−99.0%, −2.0%)	0.011	0.022
PCoA Axis 4				
IYCF	17/53	−3.5% (−99.0%, 23.0%)	0.794	0.908
PC4		0.3% (−99.0%, 6.0%)	0.923	0.923
PC4-by-IYCF^d^		5.8% (−99.0%, 13.4%)	0.131	0.174

PD, prevalence difference (in percentage points); 95% CI, 95% confidence interval; IYCF, infant and young child feeding.

a Covariates include birthweight, infant sex, age at the 6 mo visit, infant LAZ at the 6 mo visit, infant diet diversity score at the 6 mo visit, and mother-infant Lewis-null secretor phenotype discordance coded as both, none, infant only or mother only.

b Number stunted/Total N included in the model. PCoA axis scores are modelled as continuous variables.

c Adjusted for multiple hypothesis testing by the Benjamini-Hochberg method.

d Prevalence difference-in-differences per 1 unit increase in PCoA axis score.

### Age and mother-infant FUT2+/FUT3− phenotype explain shifts in microbiome composition and *B. longum*

To investigate sources of variation in infant intestinal microbiome composition and derive interpretable measures of species turnover for use in our regression models (see previous section), we performed β-diversity analyses using PCoA constrained by infant characteristics known to be associated with microbiome composition. Infant age at stool collection explained 12.6% (p = 0.001) of the variability in microbiome composition (Table S9). Notably, EBF at 3mo was not significantly associated with microbiome composition (variance explained = 0.5%, p = 0.483) (Table S9). However, 93.2% of mothers reported exclusive breastfeeding (Table S1). Thus, there may not have been sufficient variability in infant breastfeeding practices in this cohort to identify EBF-associated differences between infant microbiomes. Also, inclusion of specimens collected throughout the 1–18 mo follow-up period in our PCoA models may have obscured associations with EBF, since EBF is only recommended up to age 6 mo.

We then fitted a multivariable constrained PCoA model that included both infant age at collection and mother-infant FUT2+/FUT3− phenotype. We retained the latter variable to capture variation in microbiota composition associated with mother-infant FUT2+/FUT3−status after controlling for infant age, because of our *a priori* aim to investigate whether microbiome variation due to mother-infant FUT2/FUT3 phenotype was a modifier of the IYCF intervention, and our finding that the *mother only* FUT2+/FUT3− group was associated with a modified intervention effect. In the full model, age explained 12.8% (p = 0.001) and mother-infant FUT2+/FUT3− phenotype explained 0.81% (p = 0.323) of the variation in microbiome composition (Table S9), indicating that infant age was the main driver of microbiome-wide changes in species composition.

Age-related maturation in species composition during the 1–18 mo postpartum period was characterized by decreased relative abundance of *B. longum*, and increased relative abundance of *Dorea longicatena*, *Dorea formicigenerans, Prevotella copri*, and *Faecalibacterium prausnitzii* (Figure S4). Mother-infant FUT2+/FUT3−discordance was characterized by increased relative abundance of *B. longum* in the *none* and *infant only* groups (Figures S5–S7). Thus axis 1 reflected infant microbiome species maturation characterized by a longitudinal shift away from dominance by *B. longum* to species more characteristic of older infants. While axis 2 reflected variability in species composition associated with mother-infant FUT2+/FUT3−phenotype that was characterized by higher *B. longum* abundance in infants with higher axis 2 scores.

### *B. longum* relative abundance modifies the effect of the IYCF intervention on stunting at 18 mo

We used multivariable regression to determine if differences in the relative abundances of specific microbiome species that characterized the constrained PCoA axes were also associated with significant effect modification of the IYCF intervention. Amongst infants randomized to the intervention, a one standard deviation increase in *B. longum* relative abundance when the intervention started was associated with 49.1% (95% CI: 24.6%, 73.6%, adjusted p = 0.002) greater prevalence of stunting at age 18 mo (Table 3) and with smaller LAZ (differences-in-differences −0.86 (95% CI: −1.20, −0.52, adjusted p < 0.001) (Table S10). No other species–level interactions with the IYCF intervention were identified (Table 3 and Table S10). Overall, our findings indicate that greater relative abundance of *B. longum* at initiation of the IYCF intervention was associated with less efficacy of the intervention to reduce stunting and increase LAZ at age 18 mo.

**Table 3:** Multivariable regression models^a^ to estimate modification of IYCF on stunting at 18 mo by infant gut microbiome species in 53 infants in whom fecal specimens were available at the 6-month follow-up visit and stunting status was ascertained at the 18-month follow-up visit.

	n/N^b^	PD (95% CI)	p-value	Adjusted p-value^c^
*Bifidobacterium longum*				
IYCF	17/53	−21.7% (−46.9%, 3.5%)	0.092	0.482
B.longum		−24.0% (−43.3%, −4.8%)	0.015	0.097
B.longum-by-IYCF^d^		49.1% (24.6%, 73.6%)	<0.001	0.002
*Bifidobacterium pseudocatenulatum*				
IYCF	17/53	−21.9% (−49.5%, 6.0%)	0.12	0.503
*B.pseudocatenulatum*		37.5% (9.7%, 65.2%)	0.008	0.082
*B.pseudocatenulatum*-by-IYCF^d^^,^^e^		−47.8% (−75.7%, −20.0%)	0.001	0.005
*Escherichia coli*				
IYCF	17/53	−1.9% (−29.1%, 25.3%)	0.889	0.966
E.coli		0.3% (−11.7%, 12.3%)	0.961	0.985
E.coli-by-IYCF^d^		−8.3% (−22.6%, 5.9%)	0.253	0.716
*Dorea longicatena*				
IYCF	17/53	−4.1% (−31.0%, 22.8%)	0.766	0.966
*D.longicatena*		−0.1% (−10.6%, 10.4%)	0.985	0.985
*D.longicatena*-by-IYCF^d^		5.0% (−32.0%, 42.0%)	0.791	0.890
*Dorea formicigenerans*				
IYCF	17/53	−17.5% (−35.9%, 0.9%)	0.062	0.434
*D.formicigenerans*		−17.1% (−33.0%, −1.2%)	0.035	0.174
*D.formicigenerans*-by-IYCF^d^		−33.6% (−79.1%, 11.8%)	0.147	0.528

PD, prevalence difference (in percentage points); 95% CI, 95% confidence interval; IYCF, infant and young child feeding.

a Covariates include birthweight, infant sex, age at the 6 mo visit, infant LAZ at the 6 mo visit, infant diet diversity score at the 6mo visit, and mother-infant Lewis-null secretor phenotype discordance coded as both, none, infant only or mother only.

b Number stunted/Total N included in the model. Species relative abundances are centered and scaled to standard deviations and modelled as continuous variables.

c Adjusted for multiple hypothesis testing by the Benjamini-Hochberg method.

d Prevalence difference-in-differences per 1 SD increase in relative abundance.

e Winsorized to the 90th percentile prior to centering and scaling.

### Mother only FUT2+/FUT3− phenotype or lower *B. longum* relative abundance prevented worse faltering

**Fig. 1:**
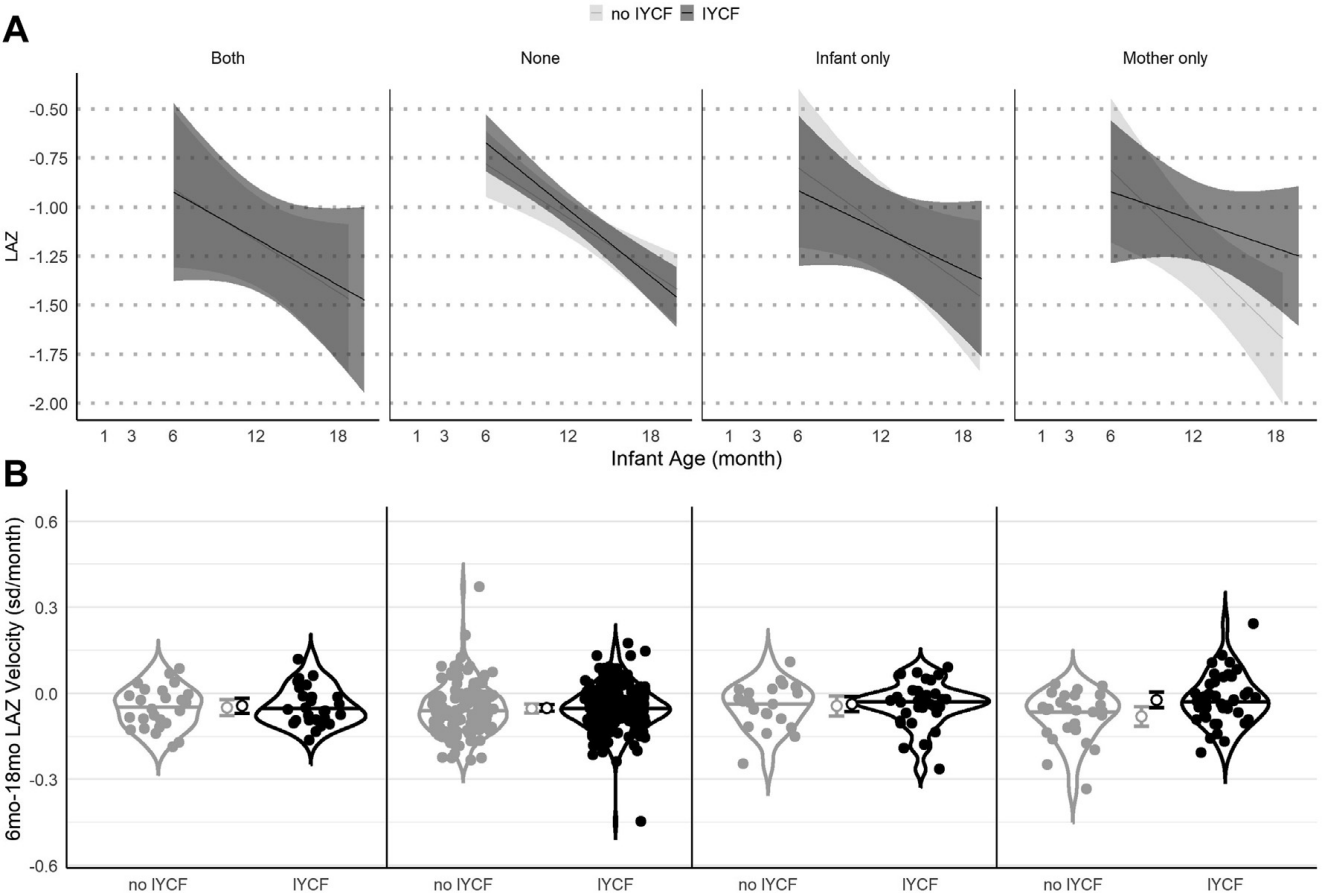
**Infant growth trajectories and velocities by IYCF and mother-infant FUT2+/FUT3-phenotype. (A)** LAZ by infant age from 1 to 18 mo (n = 792), stratified by IYCF (light grey, no IYCF; dark grey, IYCF) and mother-infant FUT2+/FUT3− phenotype. Lines illustrate average trajectories. Shaded areas are 95% confidence bands. **(B)** Violin plots of LAZ velocity from 6 to 18 mo of age, stratified by IYCF (light grey, no IYCF; dark grey, IYCF) and mother-infant FUT2+/FUT3− phenotype. Open circles with bars indicate mean LAZ velocity and 95% CIs.

To investigate whether these reductions in the efficacy of the IYCF intervention were due to differences in linear growth restriction or growth velocity, we plotted (i) LAZ growth trajectories and (ii) LAZ velocities from 6 to 18 mo of age (sd/mo), by both randomization to the IYCF intervention and paired mother-infant FUT2+/FUT3− phenotype. Infants in the *mother only* group randomized to IYCF, who benefitted more from the IYCF intervention in our interaction models, had a less steep decline in LAZ on average compared to infants randomized to no IYCF (Fig. 1). In addition, although all groups of infants had negative 6–18 mo velocities on average, infants in the *mother only* group who were randomized to IYCF had higher velocity (−0.02 sd/mo, 95% CI: −0.05, 0.00) compared to those in the no IYCF group (−0.08 sd/mo, 95% CI: −0.11, −0.05) (Fig. 1).

**Fig. 2:**
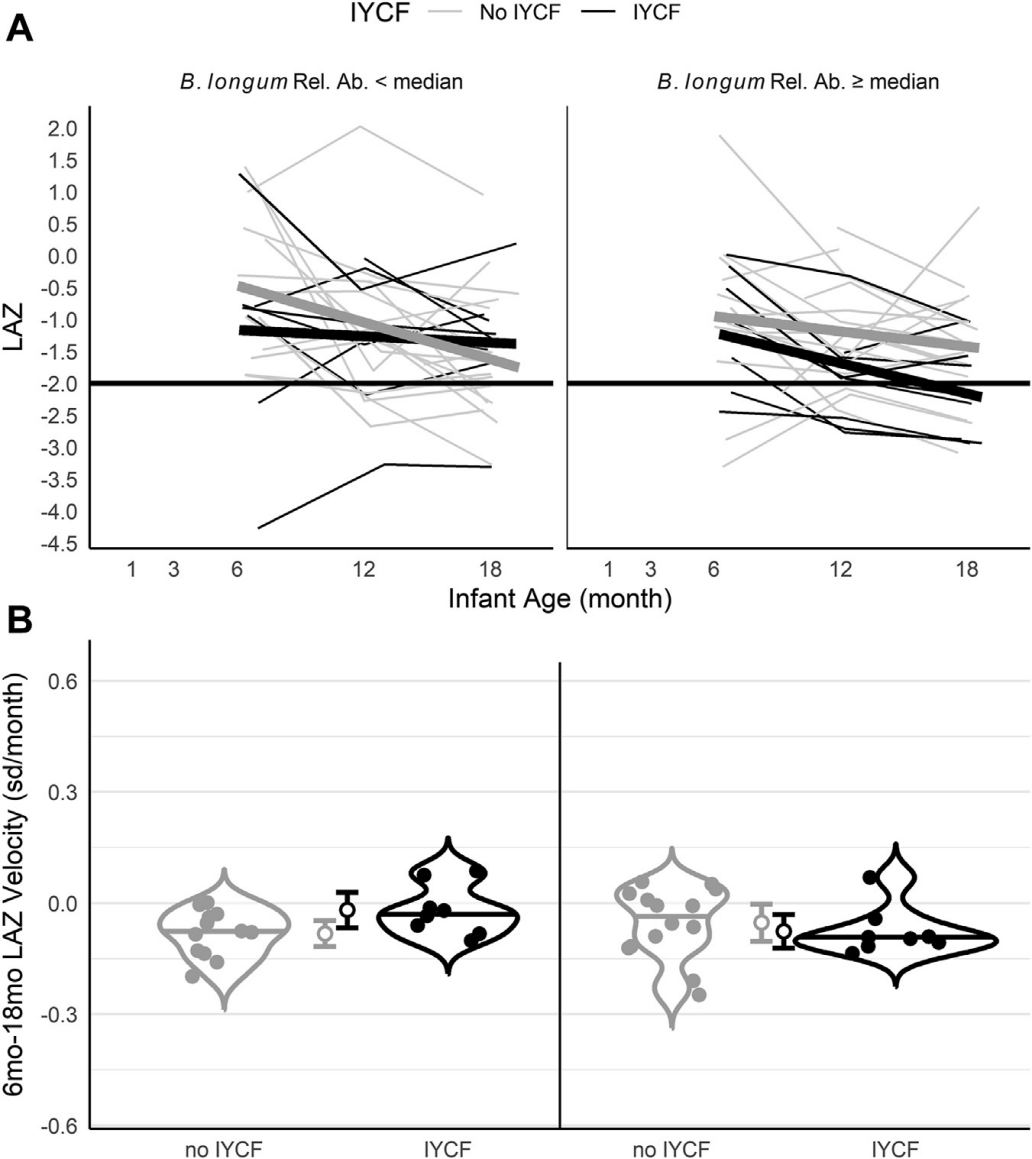
**Infant growth trajectories and velocities by IYCF and infant gut *Bifidobacterium longum* relative abundance. (A)** LAZ by infant age from 1 to 18 mo (n = 53), stratified by IYCF and *Bifidobacterium longum* relative abundance (light grey, ≤median relative abundance; dark grey, >median relative abundance). Thin lines illustrate individual trajectories. Thick lines show average trajectories. **(B)** Violin plots of LAZ velocity from 6 to 18 mo of age, stratified by IYCF (light grey, no IYCF; dark grey, IYCF) and mother-infant FUT2+/FUT3− phenotype. Open circles with bars indicate mean velocity and 95% CIs.

We repeated these analyses to compare LAZ trajectories and velocities by both IYCF arm and *B. longum* relative abundance at the 6mo visit. *B. longum* relative abundance was included in our regression models as a continuous variable, but to facilitate visualization we stratified LAZ trajectories and velocities above or below the median *B. longum* relative abundance. Among infants below the median, those who were randomized to no IYCF had declining growth trajectories on average, while infants randomized to the IYCF intervention did not (Fig. 2). Furthermore, among infants below the median, the mean 6–18 mo velocity was negative in both intervention arms, but trended toward being higher in the group randomized to the IYCF intervention (−0.02 sd/mo, 95% CI: −0.07, 0.03) compared to those randomized to no IYCF (−0.08 sd/mo 95% CI: −0.12, −0.05); however, the 95% CIs overlapped. In contrast, among infants above the median relative abundance, both intervention arms had declining growth trajectories (Fig. 2) with similar negative average velocities (Fig. 2).

Overall, these results indicate that infants in the *mother only* FUT2+/FUT3− group and infants with lower *B. longum* relative abundance at 6 mo were spared from more severe LAZ declines when provided with the IYCF intervention, which contributed to a lower probability of stunting at 18 mo despite some degree of growth faltering in each group.

### *B. longum* strains most similar to subspecies *infantis* dominate the early infant gut microbiome

**Fig. 3:**
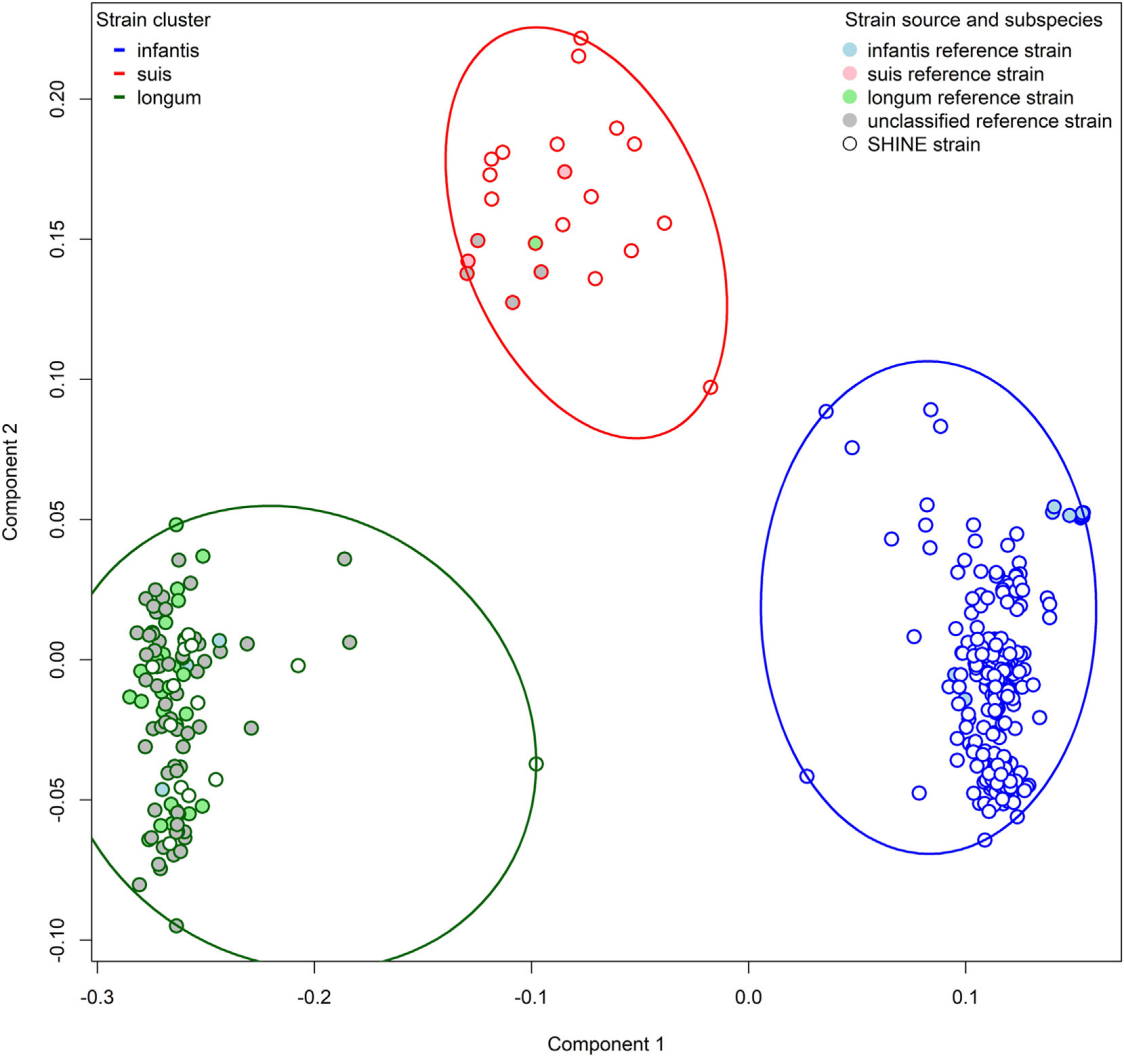
***Bifidobacterium longum* strain clusters.** Ordination plots of PCoA of Jaccard dissimilarities between PanPhlan3.0 pangenome profiles of the dominant *Bifidobacterium longum* strain in each fecal specimen (N = 284). Three clusters were identifiable. Individual strains are indicated by small circles. Strains in the same cluster are enclosed by a large ellipse and are differentiated by color (blue, *B. infantis*; dark green, *B. longum longum*; red, *B. suis*). Filled small circles indicate reference strains (light blue, *B. infantis*; pink, *B. suis*, light green, *B. longum longum*; grey, unclassified). Open small circles indicate SHINE strains.

Next, we aimed to identify whether infants carried different strains of *B. longum* using a pangenome approach. To assess similarities between SHINE *B. longum* strains and previously characterized subspecies, we included the 118 reference strains provided with PanPhlan3.0. We performed PCoA using Jaccard dissimilarities between gene family profiles and identified three clusters of *B. longum* strains by visualization of ordination plots (Fig. 3 and Figure S8). We, therefore, performed hierarchical clustering of these Jaccard dissimilarities to place strains into three distinct clusters (Figure S8). Subspecies *infantis* reference strains (15 strains) predominantly grouped with the largest cluster (hereafter called the *B. infantis* cluster) which included 255 SHINE strains (Fig. 3 and Figure S8). Subspecies *longum* reference strains (29 strains) predominantly grouped with the second largest cluster (*B. longum longum* cluster), along with 3 *B. infantis*, 64 *unclassified* and 14 SHINE strains. The remaining cluster included two *B. suis*, one *B. longum longum*, 4 *unclassified* subspecies reference strains and 15 SHINE strains (*B. suis* cluster) (Fig. 3 and Figure S8).

### Infant microbiomes shift away from a predominance of *B. longum* strains in the subspecies *infantis* cluster

To investigate predictors of *B. longum* relative abundance in infants over time, we fitted a longitudinal multivariable zero-inflated mixed-effects model. Infant age and mother-infant FUT2+/FUT3− phenotype were significant predictors of longitudinal *B. longum* relative abundance. Each one month increase in age was associated with a 0.91-fold decrease (95% CI: 0.90, 0.92) in *B. longum*, and the *mother only* FUT2+/FUT3− group had the lowest relative abundance of *B. longum* throughout follow-up (Figure S11) with a 0.71-fold decrease in relative abundance (95% CI: 0.59, 0.87) compared to the *both* group (Table S11).

Also, to determine predictors of *B. longum* strain cluster detection over time, we fitted multivariable longitudinal logistic regression models. Results were consistent with predictors of *B. longum* relative abundance. Each one month increase in age was associated with a 0.85-fold decreased odds (95% CI: 0.75, 0.96) of the *B. infantis* cluster over time (Table S12). At the same time, the *B. suis* and *B. longum longum* clusters increased with age (Table S12 & Figure S12). Female infants had 8.35-fold increased odds of the *B. infantis* cluster (95% CI: 3.17, 21.97); and by FUT2+/FUT3−phenotype, the *infant only* group had the highest probability of the *B. infantis* cluster throughout follow-up (Figure S13A), with a 4.66-fold increased odds (95% CI: 1.05, 20.71) (Table S12). By contrast, the *mother only* FUT2+/FUT3− group had low probability of carrying an *B. infantis* cluster strain (Figure S13A & Table S12) and the highest probability of carrying a *B. suis* cluster strain throughout follow-up (Figure S13C).

In summary, infant age and mother-infant FUT2+/FUT3− phenotype were important determinants of both *B. longum* relative abundance and strain carriage, longitudinally. *B. longum* decreased with over time and was lowest among infants in the *mother only* FUT2+/FUT3− group. The *B. infantis* cluster, which included the dominant *B. longum* strains, also decreased with over time and were less likely to be detected in the *mother only* group.

### Strains in the *B. longum* subspecies *infantis* cluster were better adapted for HMO metabolism and siderophores biosynthesis

**Fig. 4:**
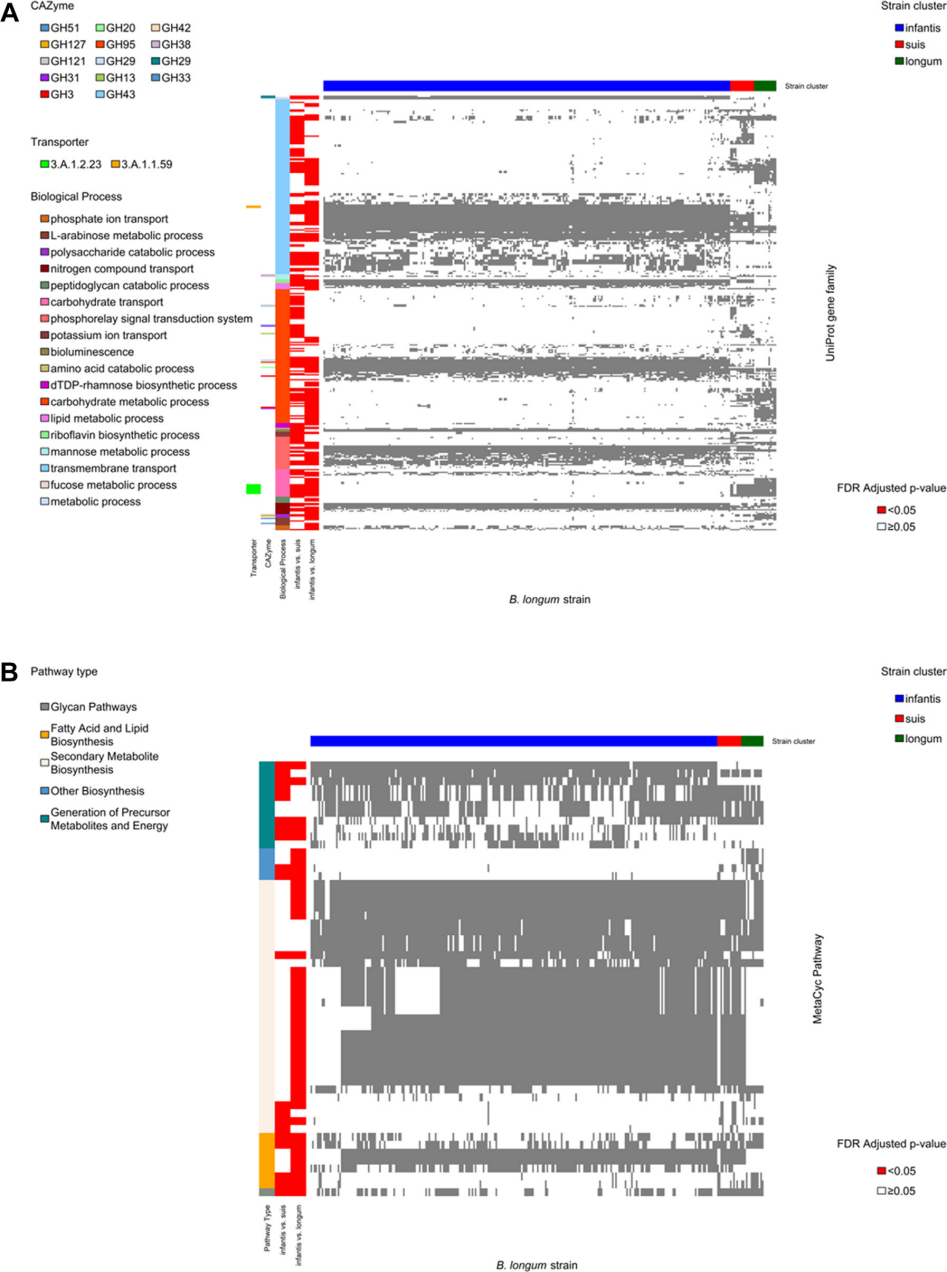
**Heatmap of differentially abundant gene families or metabolic pathways between SHINE infant *Bifidobacterium longum* strains clusters. (A)** Heatmap of UniProt gene family presence in SHINE *Bifidobacterium longum* strains (N = 284). Grey indicates UniProt gene family presence. The horizontal bar at the top indicates strain cluster (blue, *B. infantis*; dark green, *B. longum longum*; red, *B. suis*). Vertical bars from left to right indicate: UniProt gene families that differed between the *B. infantis* and *B. longum longum* cluster (red); UniProt gene families that differed between the *B. infantis* and *B. suis* cluster (red); Biological Process GO groups; CAZymes; and Transporter class. Only gene families that differed by two-sided Fisher's Exact test and with evidence of overrepresentation in a Biological Process, CAZyme or Transporter class by one-sided Fisher's Exact Test after FDR correction are presented. **(B)** Heatmap of MetaCyc pathway presence in SHINE *Bifidobacterium longum* strains (N = 284). Heatmap colors, horizontal and vertical bars are as for panel A. Only pathways that differed by two-sided Fisher's Exact test and with evidence of overrepresentation in a pathway type by one-sided Fisher's Exact Test after FDR correction are presented.

To identify differences in metabolic potential between strain clusters, we used two-sided Fisher's Exact tests to compare UniProt gene family presence between clusters. We then performed overrepresentation analyses^43^ of differentially frequent gene families by one-sided Fisher's Exact test to determine whether they were more likely to be involved in specific GO biological processes,^46^ or to function as specific carbohydrate-active enzymes (CAZymes)^44^ or transporters.^45^ The *B. infantis* cluster was more likely to carry gene families involved in HMO degradation, including genes that function as CAZyme Glycoside Hydrolase Family 20 (GH20) (including lacto-N-biosidases), GH29 (fucosidases), GH95 (fucosidases), and GH33 (sialidases) (Fig. 4A, Table S13). Conversely, the other clusters were more likely to carry gene families involved in degradation of plant-derived polysaccharides, including GH42 (β-galactosidases), GH51 (L-arabinfuranosidases) and GH127 (β-l-arabinofuranosidase) (Fig. 4A, Table S13). Similarly, the *B. infantis* cluster was more likely to have gene families that function in oligosaccharide uptake (TCID 3.A.1.1.59), but was less likely to have gene families involved in uptake of fructose and other sugars (TCID 3.A.1.2.23) (Fig. 4A, Table S13).

We also identified differences between strain clusters in the frequency of 115 MetaCyc pathways^33^ (Figure S10). Pathways that were more common in the *B. infantis* cluster were involved in generation of precursor metabolites and energy, and secondary metabolite biosynthesis (which predominantly included pathways for biosynthesis of siderophores and antimicrobials) (Fig. 4B, Table S13). Conversely, the *B. infantis* cluster was less likely to include pathways involved in glycan metabolism and polymeric compound degradation (e.g. pectin, xylan, and arabinogalactan degradation pathways), and in fatty acid and lipid biosynthesis (Fig. 4B, Table S13).

Overall, infant gut microbiomes were dominated by *B. longum* strains that were most similar to *B. infantis* in their UniProt gene family profiles. Strains in the *B. infantis* cluster had greater capacity for HMO degradation and uptake of oligosaccharides than plant-derived polysaccharides, as well as greater capacity for siderophore and antimicrobial production.

## Discussion

In this study of HIV-unexposed infants enrolled in the SHINE trial in rural Zimbabwe, we tested the hypothesis that mother-infant FUT2/FUT3 phenotype and infant gut microbiome modify the effect of an intervention that included SQ-LNS to improve IYCF, on infant stunting and LAZ at age 18mo. Our goal was to define mechanisms that explain the rather modest effects of interventions containing SQ-LNS on chronic undernutrition, reasoning that maternal-infant HBGA phenotypes might be important given their combined role in shaping the early-life microbiome. We found the following features are associated with greater reduction in stunting at 18 mo by the IYCF intervention: (i) discordance in mother-infant FUT2+/FUT3− phenotype, where the mother has the phenotype but the infant does not; (ii) changes in microbiome species composition that reflected a shift from a *B. longum*-dominant microbiome to a microbiome with less *B. longum* and a greater abundance of species characteristic of older infants; and (iii) decreased *B. longum* abundance that was associated with paired mother-infant FUT+/FUT3− status.

The *mother only* FUT2+/FUT3− group showed evidence of greater reduction in stunting following receipt of one year of the intervention. The *mother only* FUT2+/FUT3− group also had lower relative abundance of *B. longum* throughout follow-up. Active maternal FUT2/FUT3 genes are key determinants of HMO composition,^14^, ^15^, ^16^ and HMO composition among FUT2+/FUT3− mothers is distinct from FUT2+/FUT3+ and FUT2− women.^14^^,^^16^ Differences in HMO composition influence growth and activity of Bifidobacterium populations in the infant gut.^11^^,^^50^ Infant fucosyltransferase phenotype may also affect gut microbiome composition,^20^, ^21^, ^22^^,^^51^^,^^52^ for example infant gut Bifidobacterium abundance and diversity differ by infant FUT2/FUT3 phenotype^21^; and CAZymes found in *B. longum* species that function in HMO degradation (GH29 and GH95) have also been found to function in host intestinal glycan degradation in infants.^53^ The evidence, therefore, suggests that the FUT2/FUT3 phenotypes of mother and infant, together, can elicit a strong prebiotic selective pressure, driven by HMO and infant glycan composition that influences Bifidobacteria and broader infant gut microbiota composition.

In our PCoA model, infant microbiome species maturation predominantly reflected decreased abundance of *B. longum* and increased abundance of *D. longicatena*, *D. formicigenerans P. copri*, and *F. prausnitzii*. Delayed gut microbiota maturation has been reported in children with severe acute malnutrition (SAM), while improvements in microbiota maturity and relative abundance of weaning-phase taxa have been correlated with improvements in acute undernutrition.^54^ Our analyses provide evidence that infant gut microbiome species maturation also increased the effect of the IYCF intervention on chronic undernutrition (stunting) at 18 mo, while greater relative abundance of *B. longum*, an age-discriminatory taxon which is associated with a younger microbiome, reduced the effect.

Utilization of HMOs by Bifidobacterium varies between species and strains.^55^, ^56^, ^57^, ^58^, ^59^, ^60^, ^61^ We identified three clusters of dominant *B. longum* strains that also varied by infant age and mother-infant FUT2+/FUT3− phenotype. The cluster of strains with pangenomes most closely resembling *B. infantis* had the highest prevalence in early infancy. However, from age 6 mo to 18− mo there was a marked decrease in detection of *B. infantis* cluster strains and a corresponding increase in detection of strains with pangenomes most similar to *B. suis,* which had the highest prevalence at 18 mo. Strains most similar to *B. longum longum* increased more slowly. This is consistent with a previous report.^62^ In that report, *B. infantis* was dominant in early infancy but decreased considerably after age 6 mo, during which period *B. suis*/*B. suillum* strains increased, peaking by age 18 mo. The transitional *B. suis*/*B. suillum* clade harbored functional capacity to degrade both HMOs and dietary polysaccharides, suggesting it may be an adaptation of the infant gut microbiome to a period when breastfeeding co-occurs with introduction of complementary foods.^62^ In this work, we show that differences between mother and infant in FUT2+/FUT3− phenotype likely play an important role in driving this transition, whereby, throughout follow-up, the *mother only* FUT2+/FUT3− group had a low prevalence of the *B. infantis* cluster and the highest prevalence of the *B. suis* cluster.

*B. infantis* are particularly well adapted for HMO utilization.^63^^,^^64^ *B. longum longum*, on the other hand, do not grow as well on HMO, but utilize diet-derived polysaccharides.^65^ In our analyses, gene families in the *B. infantis* cluster were more likely to be involved in HMO degradation and uptake. Two CAZyme families more likely to be carried by the *B. infantis* cluster (GH29^66^ and GH33^57^) were previously reported as more prevalent in *B. infantis* strains. In contrast, in the other clusters, gene families were more likely to be involved in uptake of fructose and other simple sugars, including the sugar transporter 3.A.1.2.23, which was previously described in a different *B. longum* subspecies.^67^ Furthermore, strains in the *B. suis* and *B. longum longum* clusters were more likely to carry pathways for pectin, xylan, and arabinogalactan degradation.

Pathways in *B. infantis* cluster strains were also more likely to be involved in siderophore and antimicrobial biosynthesis. Heavy metals such as iron and zinc are essential minerals for nearly all bacteria and their mammalian hosts. Strategies utilized by bacteria to acquire heavy metals include biosynthesis of low molecular weight iron-chelating compounds, called siderophores, to scavenge iron and other essential metals such as zinc.^68^ Bifidobacterium species isolated from iron-deficient children efficiently sequester iron via siderophore production,^69^ which may provide a competitive advantage to Bifidobacteria,^70^ and along with antimicrobial biosynthesis,^71^ may also help protect the infant from enteric pathogens that require essential metals for colonization. However, essential metals such as iron and zinc are key components in SQ-LNS formulations,^72^, ^73^, ^74^ and produce improvements in linear growth and reductions in stunting.^75^^,^^76^ These findings suggest potential mechanisms by which a “younger” microbiome may constrain the beneficial effects of an SQ-LNS intervention on infant stunting. However, siderophore activity varies considerably between Bifidobacterium species and strains, and no research to date has investigated sequestration of essential metals by *B. infantis* strains commonly found in resource-limited settings. More research is required to fully elucidate the biological mechanisms and downstream pathways to stunting that are involved.

Our results are in contrast to a previous RCT that investigated infant microbiome composition as a modifier of SQ-LNS impact on linear infant growth in Malawi.^77^ However, in the primary analyses of that RCT, there was no effect of SQ-LNS on linear growth.^78^ Furthermore, SQ-LNS was provided to both mothers during pregnancy and infants starting at 6 mo postpartum, active control interventions (iron-folate or multiple micronutrient supplements) were provided to mothers^78^ and 16S rRNA gene amplification and sequencing were used to characterize the microbiota which can affect both taxon detection (including Bifidobacterium) and study results.^79^ In addition, the SHINE IYCF intervention also included a behavioral change intervention which focused on increasing nutrient density and dietary diversity, the importance of locally available foods for infant health, and the introduction of SQ-LNS.^80^ However, prior formative work in Zimbabwe identified low dietary diversity to be the predominant feeding problem, and we included an indicator in our models of whether infants' diets met minimum dietary diversity to control for differences between infants. Furthermore, in previous work from SHINE (not yet published) the biggest contributor to closing nutrient gaps in infants’ diets following the IYCF intervention was the SQ-LNS component of the package. Nevertheless, changes in consumption of specific nutrient-rich foods could potentially contribute to the effect of the SHINE intervention in addition to SQ-LNS. By contrast, results from a more recent RCT were consistent with our findings, which found that suppression of *B. longum* by amoxicillin allowed the gut microbiota of children with SAM to better adapt to a solid-food diet by reducing the abundance of taxa specialized for breast milk utilization, resulting in improved anthropometric indicators of infant nutritional status.^81^

However, it is essential to note that *B. infantis* is a critical early infant gut bacteria with important benefits for infant health.^82^ In addition, *B. infantis* improved ponderal (weight) growth when administered to infants ∼4 mo of age with SAM in two RCTs.^83^^,^^84^ However, the effects on chronic undernutrition were not statistically significant. Our findings suggest that a shift away from a persistently “less mature” microbiome that is characterized by a high abundance of *B. longum* and carriage of strains that are better suited to HMO metabolism, may also be critical to support efficacy of nutrient supplements which start with the introduction of complementary feeding, to support linear growth and reduce chronic infant undernutrition. Furthermore, we identify drivers of early infant *B. longum* abundance and strain carriage which, if further elucidated, may be employed to shape the infant microbiome into a more favorable composition to optimize the beneficial effects of interventions that utilize SQ-LNS on chronic undernutrition at this critical period in infancy.

There are some limitations of this work. First, from our cohort of 1169 HIV-unexposed infants, we had microbiome data from 172 infants. Importantly, infants included in our analyses were comparable to excluded infants (Table S1). Also, our results are biologically consistent as illustrated by our models in which mother-infant FUT2+/FUT3− phenotypes predicted both *B. longum* relative abundance and strain cluster detection. The consistently significant interactions for mother-infant FUT2+/FUT3− phenotypes, *B. longum* abundance (which mother-infant FUT2+/FUT3− phenotypes predicted), and microbiome maturation (which was characterized by *B. longum* abundance) in different subsets of our cohort increase the internal validity of our results. In addition, our interaction p-values were very small, even after adjustment for multiple testing, and we assessed interaction on the risk difference scale, which has been shown to produce only a small elevation in the false discovery rate in analyses of interaction that use very small sample sizes.^38^ That said, our sample size may have limited power to detect effect modification by other taxa that are indicative of a “more mature” post-weaning microbiome such as *P. copri* and *F. prausnitzii*, and larger studies are needed to corroborate these findings and produce more precise estimates of effect modification. Our analyses were not able to investigate effect modification by actual microbial metabolic activity, maternal HMO composition or infant gut glycobiome, which would require multi-omics approaches such as metatranscriptomics and metabolomics.

In conclusion, we present analyses of moderators of IYCF impact on infant stunting at 18 mo in an RCT using SQ-LNS in rural Zimbabwe. We report that (i) infant microbiome species maturation, characterized by a shift from *B. longum* dominance, particularly *B. longum* strains that are most similar to the proficient HMO utilizer *B. infantis*, was associated with increased IYCF reduction of stunting at age 18 mo; (ii) *B. longum* relative abundance was associated with reduced IYCF benefits on stunting; and (iii) discordance in mother-infant FUT2+/FUT3− phenotype, where the mother had the phenotype but the infant did not, was also associated with increased IYCF reduction of stunting and predicted reduced *B. longum* relative abundance and strain carriage. Future work should investigate how variations in maternal HMOs and infant gut glycans determine gut Bifidobacterium species, strain composition and microbiome maturation, to inform development of next generation interventions that harness *B. longum* strains, HMOs and glycans in early life to induce adequate maturation of the microbiome, which when used in combination with nutrient supplements, will maximize their efficacy, reduce chronic undernutrition, and optimize growth at this critical period in life.

## Contributors

ARM, MNNM, AJP conceptualized and designed the study. KM, RN, BC, FDM, NT, JT, BM collected data and biospecimens. HMG, IB, SKG, RCR, FF, LC processed fecal specimens. KM conducted laboratory analyses to ascertain FUT2 and FUT3 status. CE, EKG and MK developed the FUT2 and FUT3 analysis plan. EKG developed and conducted the microbiome statistical analysis plan. TJE, EKG conducted bioinformatics. ARM, EKG analyzed and interpreted the data. EKG wrote the original manuscript draft. ARM and AJP supervised and verified the data. All authors read and approved the final version of the manuscript.

## Data sharing statement

The raw metagenome sequencing data generated in this study have been deposited in the European Bioinformatics Database under accession code PRJEB51728. Epidemiologic data files and final processed and annotated metagenome sequencing data files (taxa) are available at https://doi.org/10.5281/zenodo.7471082. Code for statistical analyses are available from the corresponding author.

## Declaration of interests

AJP was supported by Wellcome Trust grant 108065/Z/15/Z. ARM was supported by Bill & Melinda Gates Foundation grant OPP1021542 and OPP1143707, with a subcontract to the University of British Columbia 20R25498 EKG was supported by The Nutricia Research Foundation grant 2021-52. T.J.E. was paid a scientific consulting fee in relation to the analysis of the data presented here by the Zvitambo Institute for Maternal and Child Health Research. RCR declares remittance from Abbott Nutrition Health Institute and Nutricia for public conference talks outside of the submitted work in the past 36 months. All other authors declare that they have no competing interests.
