# Supplementary material for: Bifidobacterium longum and microbiome maturation modify a nutrient intervention for stunting in Zimbabwean infants
Source: eBioMedicine. 2024 Sep 27;108:105362. doi: 10.1016/j.ebiom.2024.105362 (PMC11467582; doi:10.1016/j.ebiom.2024.105362)
Supplement: Supplemental Information [file mmc1.docx]

**Supplementary Material**

Supplementary methods

Figure S1. Flow chart of pathways for histo-blood group antigen synthesis by functional FUT2 and FUT3 status.

Figure S2. Flow of participants through the SHINE trial and the analyses reported in this study.

Figure S3. Ordination plots of principal coordinates analyses constrained by infant age and mother-infant FUT2+/FUT3- phenotype.

Figure S4. Species loadings for constrained principal coordinates analyses axis 1 (infant age).

Figure S5. Species loadings for constrained principal coordinates analyses axis 2 (mother-infant FUT2+/FUT3- phenotype).

Figure S6. Species loadings for constrained principal coordinates analyses axis 3 (mother-infant FUT2+/FUT3- phenotype).

Figure S7. Species loadings for constrained principal coordinates analyses axis 4 (mother-infant FUT2+/FUT3- phenotype).

Figure S8. Heatmap of UniProt gene family presence in *Bifidobacterium longum* strains.

Figure S9. Differences in UniProt gene family profiles between SHINE infant *Bifidobacterium longum* strain clusters.

Figure S10. Differences in MetaCyc pathway profiles between SHINE infant *Bifidobacterium longum* strain clusters.

Figure S11. Relative abundance of *Bifidobacterium longum* in the infant gut microbiome over time

Figure S12. Probability of *Bifidobacterium longum* strain cluster detection over time.

Figure S13. The dominance of *Bifidobacterium longum* strains most similar to *B. infantis* varies by mother-infant FUT2+/FUT3- phenotype.

Table S1. Baseline characteristics of infants in this substudy are comparable to the wider SHINE trial cohort

Table S2. Distribution of mother and infant FUT2 and FUT3 status and combinations

Table S3. Distribution of paired mother and infant FUT2 and FUT3 phenotype combinations

Table S4. Number stunted / Total N per subgroup by IYCF and mother-infant FUT2 and FUT3 phenotype combinations used in the multivariable regression models to determine modification of the IYCF intervention effect on stunting at 18mo by mother-infant FUT2 and FUT3 phenotypes which are presented in Table 1 and Table S5.

Table S5. Multivariable regression model to estimate modification of IYCF on stunting at 18mo by mother-infant discordance in various FUT2 and FUT3 phenotype combinations among 610 infants in whom mother and infant FUT2 and FUT3 status was ascertained

Table S6. Multivariable regression model to estimate modification of IYCF on LAZ at 18mo by mother-infant discordance in various FUT2 and FUT3 phenotype combinations among 610 infants in whom mother and infant FUT2 and FUT3 status was ascertained

Table S7. Number stunted / Total N per subgroup by IYCF and mother-infant FUT2 and FUT3 phenotype combinations used in the multivariable regression models to determine modification of the IYCF intervention effect on LAZ at 18mo by mother-infant FUT2 and FUT3 phenotypes which are presented in Table S6.

Table S8. Multivariable regression models to estimate modification of IYCF on LAZ at 18mo by infant gut microbiome species turnover in 53 infants

Table S9. Analysis of Variance for Distance Matrices (ADNOIS2) using Bray-Curtis Dissimilarities to identify infant characteristics that significantly explain variation in microbiome composition

Table S10. Multivariable regression models to estimate modification of IYCF on LAZ at 18mo by infant gut microbiome species in 53 infants

Table S11. Multivariable mixed-effects zero-inflated beta regression to identify predictors of Bifidobacterium longum relative abundance

Table S12. Multivariable logistic regression to identify predictors of Bifidobacterium longum strain cluster prevalence

Table S13. Overrepresentation of UniProt gene families and Metacyc pathways, which differ between strain clusters, by GO biological process, CAZyme or Transporter class or Metacyc pathway type

Supplemental Data file 1 includes Table S13

**Supplementary methods**

**Study** **Design**

The Sanitation Hygiene Infant Nutrition Efficacy (SHINE) trial was a 2x2 factorial cluster-randomized trial that enrolled 5280 pregnant women at a median age of 12.5 weeks gestation between November 2012 and March 2015 to test the impact of improved household water quality, sanitation, and hygiene (WASH) and improved infant and young child feeding (IYCF) via provision of SQ-LNS to the infant from age 6mo-18mo, on linear growth and anemia at age 18mo. A detailed description of the SHINE trial design and methods has been published(1,2).

Briefly, research nurses made home visits twice during pregnancy and at infant ages 1, 3, 6, 12, and 18 months. At baseline, maternal education and age, household wealth(3), existing water and sanitation services, and household food security(4) were assessed, and mothers were tested for HIV via a rapid testing algorithm. Infant birth date, weight, and delivery details were transcribed from health facility records. Infant weight and length were measured at every postnatal visit. Nurses were standardized against a gold-standard anthropometrist every 6 months, with retraining provided to those who failed to meet predefined criteria.

Part-way through the trial (from mid-2014 onwards) mother-infant pairs were invited to join a substudy to collect additional biological specimens. Women were informed about the substudy at their 32-week gestation visit and those with live births were enrolled at the 1-month postnatal visit, or as soon as possible thereafter.

**Specimen collection and processing**

Mothers collected fecal specimens prior to the research nurse visit. Fecal specimens were placed in a cold box and transported to the field laboratory, where they were stored at -80°C until transfer to the central laboratory in Harare for long-term archiving at -80°C, with generator back-up. Fecal specimens were transferred via private courier on dry ice from Harare, Zimbabwe to Vancouver, British Columbia for metagenomic analyses.

The Qiagen DNeasy PowerSoil Kit was used to extract total DNA from 200mg of feces, according to manufacturer’s instructions. Paired-end libraries were constructed using the Illumina TruSeq kit and using New England Biosystem TruSeq compatible library preparation reagents. Libraries were sequenced at the British Columbia Genome Sciences Centre using the Illumina HiSeq 2500 platform. Forty-eight libraries were pooled and included per sequencing lane. Negative controls were included to capture microbial contamination in the DNA extraction and library preparation steps.

The analyses presented here utilize data and specimens from HIV-uninfected mothers and their infants enrolled in the specimen collection substudy. The fecal microbiome was characterized in 354 specimens collected from 172 HIV-unexposed infants from 1 to 18mo of age. A mean(sd) of 2.0(1.0) samples were analyzed per child. Specimen selection for inclusion into the current microbiome study was conducted to enhance longitudinal profiling of the mother and infants gut microbiota. Of the mother-infant pairs within the substudy, those with at least one maternal fecal specimen (of 2 possible) and at least 2 infant fecal specimens (of 5 possible) were included in the gut microbiome analyses. An additional 94 samples collected at the 1 and 3-month visits [2 specimens], that did not meet these criteria, but had microbiome sequencing data available from a separate study examining rotavirus vaccine immunogenicity in the SHINE trial, were also included in these analyses. Infants included in these analyses largely resembled the population of live-born infants from the wider SHINE trial who were not included in these analyses (Table S1). However, infants in the current analyses had slightly older mothers and longer gestational ages, and fewer were born during the hungry season, but more were in a household that met the minimum dietary diversity score (Table S1). Overall, the majority were born by vaginal delivery (91.6%) in an institution (91.5%) and were exclusively breastfed (83.2% at 3 months). The prevalence of stunting was 26.9% at 18mo (Table S1). Mean[min, max] follow-up was 18.2months [16.8, 20.7]

**Bioinformatics**

Sequenced reads were trimmed of adapters and filtered to remove low-quality, short (<60 base-pairs), and duplicate reads, as well as those of human, other animal or plant origin using KneadData with default settings. Overall, 354 unique whole metagenome sequencing datasets were used from fecal specimens collected from 1mo to 18mo from 172 infants with available mother and infant FUT2 and FUT3 phenotypes (Figure S2). On average, 10.8±3.7 million paired end quality-filtered reads were generated per sample. Assessment of negative controls and technical variation have been previously reported(5). Species composition was determined by mapping reads to clade-specific markers using MetaPhlAn3.0 with default settings(6). Bacterial species and pathway abundance estimates were normalized to relative abundances. UniProt gene family profiles were generated for dominant *B. longum* strains in fecal metagenomes with sufficient coverage for pangenome analysis using PanPhlan3.0(6). To facilitate interpretation of UniProt gene family profiles, the minimum set of biological pathways sufficient to explain the gene families identified in each strain was determined using MinPath with default settings(7) and the MetaCyc database(8).

**Assessment of FUT2 and FUT3 status**

Saliva samples were collected by oral swab from mothers and infants. Available saliva from any follow-up visit was selected to assess FUT2 and FUT3 status. Secretor versus non-secretor (FUT2) and Lewis-positive versus Lewis-null (FUT3) status were ascertained for infants and their mothers using a previously reported phenotyping assay(9). We defined FUT2 and FUT3 phenotype combinations as FUT2-/FUT3+ (Lewis-positive non-secretors), FUT+/FUT3+ (Lewis-positive secretors), or FUT2+/FUT3- (Lewis-null secretors) (Table S2) according to the histo-blood group antigen synthesis pathways defined in Figure S1. Paired mother-infant phenotypes were defined as presented in Table S3.

Of 1169 HIV-uninfected mother-infant pairs in the specimen collection substudy, 999 (85.4%) mothers and 1104 (94.4%) infants were tested. Of these, FUT2 or FUT3 status could be determined in 889 (76.0%) and 999 (85.4%) mothers and infants, respectively (Table S2). Of those whose FUT2 or FUT3 status could be ascertained, FUT2+ was the most frequent FUT2 phenotype (88.1% and 85.6% in mothers and infants respectively), while FUT3+ was the most frequent FUT3 phenotype (76.8% and 74.9% in mothers and infants, respectively) (Table S2). Most mothers and infants were FUT2+/FUT3+ (60.5% and 64.9%, respectively), followed by FUT2+/FUT3- (25.1% and 23.2%, respectively), and FUT2-/FUT3+ (14.4% and 11.9%, respectively) (Table S3). The distribution of FUT2/FUT3 phenotypes were consistent with estimates from other countries in that FUT2+/FUT3+ (55% to 77%) is the most common phenotype, followed by FUT2+/FUT3- (5.7% to 25.0%) and FUT2-/FUT3+ (0.0% to 25.7%)(10,11), and are similar to reports from other countries in the Africa(12,13). However, phenotype frequencies vary by ethnicity and geographic region of the world(10,11).

**Statistical Analysis**

**β*-diversity analyses***

To explore sources of variation in microbiome composition and derive interpretable measures of microbiome species turnover, we identified infant characteristics that explain species β-diversity using constrained principal coordinates analysis (PCoA) of Bray-Curtis dissimilarities between 354 metagenomes collected from 172 infants throughout follow-up (*capscale*)(14). Characteristics of interest included factors that are known to be correlated with microbiome composition (infant age, sex, EBF at 3mo, dietary diversity, maternal and infant FUT2 and FUT3 phenotype). Statistical significance was tested by PERMONOVA of distance matrices with 1000 permutations (*adonis2*)(15). We then developed a final multivariable constrained PCoA model that included covariates which explained a significant fraction of the variance in microbiome composition (infant age at specimen collection, p<0.05), as well as mother-infant FUT2+/FUT3- discordance given our *a prior* hypothesis that FUT2 and FUT3 phenotypes are important determinants of infant microbiome composition and our finding that the mother-infant FUT2+/FUT3- phenotype was an important modifier of IYCF efficacy to reduce stunting at 18mo. Constrained PCoA axis scores in the final multivariable model represented changes in microbiome composition (species turnover) along gradients defined by each infant characteristic included in the full model.

***Biological interaction analyses***

We assessed interaction between randomization to IYCF and mother-infant FUT2/FUT3 phenotype or microbiome composition on the additive risk difference scale, which is appropriate for statistical estimation of synergistic biological effects(16) and for controlling the false discovery rate with small sample sizes(17). The primary outcome was stunting at 18mo and LAZ at 18mo was the secondary outcome. We fitted separate models for stunting and LAZ at 18mo. We used generalized linear models (*glm*) with a Gaussian distribution, an identity link, and sandwich standard errors (*sandwich*)(18) that included a term for interaction between IYCF and (i) each FUT2/FUT3 phenotype, (ii) each PCoA axis score from our final multivariable constrained PCoA model, and (iii) each species of interest. Species of interest were those which were strongly associated with PCoA axis 1 to 4 scores (loadings > 0.5 or < -0.5). Models also included IYCF, infant sex, mother-infant FUT2+/FUT3- discordance, infant age at specimen collection, an indicator of whether infants met minimum dietary diversity, and LAZ at specimen collection. We did not include WASH arm because, in prior analyses, the SHINE WASH intervention did not affect stunting or LAZ at 18mo(1) nor infant gut microbiome composition(5). Since the IYCF intervention started at the 6mo follow-up visit, our interaction models used covariate data from that visit. Likewise, while our final constrained PCoA model used all sequenced specimens collected during follow-up, we used the derived PCoA axis scores corresponding to the 6mo visit in our interaction models. We fitted a separate model for each interaction of interest. P-values were adjusted for multiple hypothesis testing to preserve the false discovery rate (FDR)(19).

***Identification and analysis of Bifidobacterium longum strain clusters***

*B. longum* strain profiles produced with PanPhlan3.0, which indicate whether UniProt gene families are present in a strain(6), were converted to Jaccard dissimilarity matrices and visualized by PCoA to ascertain the existence of strain clusters (*capscale*). Three clusters were identified, and strain cluster membership was determined by hierarchical clustering of Jaccard dissimilarities and Ward’s error sum of squares algorithm (*hclust*)(20). Hierarchical clustering dendrograms were cut at a height to obtain three clusters, as determined by visualization of PCoA ordination plots.

Differences in UniProt gene family profiles between the three *B. longum* strain clusters were determined by two-sided Fisher’s Exact test (*fisher.test*), with adjustment for multiple hypothesis testing to preserve the false discovery rate(19), and were visualized using heatmaps (*heatmap3*). 3260 UniProt gene families were differentially present between strain clusters after FDR-adjustment. We performed overrepresentation analyses(21) using one-sided Fisher’s Exact tests (*fisher.test*) to determine whether the differentially present gene families were more likely to function as particular CAZymes(22), transporters(23), or in specific GO biological processes(24). These analyses were repeated using the biological pathways determined with MinPath(7). 115 pathways were differentially present between strain clusters after FDR-adjustment. We performed overrepresentation analyses of these pathways to determine whether they were more likely to have particular biological functions defined by MetaCyc pathway types(8).

***Predictors of Bifidobacterium relative abundance and strain detection***

Predictors of *B. longum* relative abundance over time were assessed using mixed-effects zero-inflated beta regression estimated by restricted maximum likelihood (*gamlss*)(25). The model included infant age at specimen collection, sex, EBF at 3mo, minimum infant dietary diversity at specimen collection, and mother-infant FUT2+/FUT3- discordance, with random intercepts (*re*) and a first order autocorrelation structure (*corCAR1*).

Predictors of *B. longum* strain cluster were assessed by logistic regression (*glm*) using an indicator of strain cluster presence as the dependent variable, with the same covariates, and sandwich standard errors. An individual model was fitted separately for each cluster. Bias corrected(26) logistic regression was used to facilitate stable parameter estimation due to separation resulting from the small sample size(27).

All statistical analyses were conducted in R version 4.2.0. PCoA and adonis2 were performed using the *vegan* package(28). Heatmaps were generated with *heatmap3*. Mixed-effects zero-inflated beta regression was performed using the *gamlss* package(29). Bias corrected logistic regression models were fitted using the *brglm2* package. Sandwich standard errors were generated with the *sandwich* package.

**References**

1. Humphrey JH, Mbuya MNN, Ntozini R, Moulton LH, Stoltzfus RJ, Tavengwa NV, et al. Independent and combined effects of improved water, sanitation, and hygiene, and improved complementary feeding, on child stunting and anaemia in rural Zimbabwe: a cluster-randomised trial. The Lancet Global Health. 2019;7(1):e132–47.

2. Sanitation Hygiene Infant Nutrition Efficacy (SHINE) Trial Team, Humphrey JH, Jones AD, Manges A, Mangwadu G, Maluccio JA, et al. The Sanitation Hygiene Infant Nutrition Efficacy (SHINE) Trial: Rationale, Design, and Methods. Clin Infect Dis. 2015 Dec 15;61 Suppl 7(Suppl 7):S685-702.

3. Chasekwa B, Maluccio JA, Ntozini R, Moulton LH, Wu F, Smith LE, et al. Measuring wealth in rural communities: Lessons from the sanitation, hygiene, infant nutrition efficacy (SHINE) trial. PLoS ONE. 2018;13(6):1–19.

4. Maxwell D, Watkins B, Wheeler R, Collins G. The Coping Strategy Index: a tool for rapid measurement of household food security and the impact of food aid programs in humanitarian emergencies. CARE and WFP; 2003.

5. Robertson RC, Edens TJ, Carr L, Mutasa K, Gough EK, Evans C, et al. The gut microbiome and early-life growth in a population with high prevalence of stunting. Nat Commun. 2023;14(1):654.

6. Beghini F, McIver LJ, Blanco-Míguez A, Dubois L, Asnicar F, Maharjan S, et al. Integrating taxonomic, functional, and strain-level profiling of diverse microbial communities with bioBakery 3. Elife. 2021;10:e65088.

7. Ye Y, Doak TG. A parsimony approach to biological pathway reconstruction/inference for genomes and metagenomes. PLoS Comput Biol. 2009;5(8):e1000465.

8. Caspi R, Billington R, Fulcher CA, Keseler IM, Kothari A, Krummenacker M, et al. The MetaCyc database of metabolic pathways and enzymes. Nucleic Acids Research. 2018;46(D1):D633–9.

9. Colston JM, Francois R, Pisanic N, Yori PP, Mccormick BJJ, Olortegui MP, et al. Effects of Child and Maternal Histo-Blood Group Antigen Status on Symptomatic and Asymptomatic Enteric Infections in Early Childhood. Journal of Infectious Diseases. 2019;220:151–62.

10. Cooling L. Blood groups in infection and host susceptibility. Clinical Microbiology Reviews. 2015;28(3).

11. King JR, Varadé J, Hammarström L. Fucosyltransferase Gene Polymorphisms and Lewisb-Negative Status Are Frequent in Swedish Newborns, With Implications for Infectious Disease Susceptibility and Personalized Medicine. J Pediatric Infect Dis Soc. 2019;8(6):507–18.

12. Magwira CA, Nndwamato NP, Selabe G, Seheri ML. Lewis a−b− histo-blood group antigen phenotype is predictive of severe COVID-19 in the black South African population group. Glycobiology. 2024;34(1):cwad090.

13. Pollock L, Bennett A, Jere KC, Dube Q, Mandolo J, Bar-Zeev N, et al. Nonsecretor Histo–blood Group Antigen Phenotype Is Associated With Reduced Risk of Clinical Rotavirus Vaccine Failure in Malawian Infants. Clinical Infectious Diseases. 2019;69(8):1313–9.

14. Legendre P, Anderson MJ. Distance-Based Redundancy Analysis: Testing Multispecies Responses in Multifactorial Ecological Experiments. Ecological Monographs. 1999;69(1):1–24.

15. Anderson MJ. A new method for non-parametric multivariate analysis of variance. Austral Ecology. 2001;26(1):32–46.

16. VanderWeele TJ. Sample Size and Power Calculations for Additive Interactions. Epidemiol Methods. 2012;1(1):159–88.

17. Schmidt AF, Groenwold RHH, Knol MJ, Hoes AW, Nielen M, Roes KCB, et al. Exploring interaction effects in small samples increases rates of false-positive and false-negative findings: results from a systematic review and simulation study. J Clin Epidemiol. 2014;67(7):821–9.

18. Naimi AI, Whitcomb BW. Estimating Risk Ratios and Risk Differences Using Regression. Am J Epidemiol. 2020;189(6):508–10.

19. Benjamini Y, Hochberg Y. Controlling the False Discovery Rate: A Practical and Powerful Approach to Multiple Testing. Journal of the Royal Statistical Society Series B (Methodological). 1995;57(1):289–300.

20. Murtagh F, Legendre P. Ward’s Hierarchical Agglomerative Clustering Method: Which Algorithms Implement Ward’s Criterion? Journal of Classification. 2014;31(3):274–95.

21. Wieder C, Frainay C, Poupin N, Rodríguez-Mier P, Vinson F, Cooke J, et al. Pathway analysis in metabolomics: Recommendations for the use of over-representation analysis. PLoS Comput Biol. 2021;17(9):e1009105.

22. Drula E, Garron ML, Dogan S, Lombard V, Henrissat B, Terrapon N. The carbohydrate-active enzyme database: functions and literature. Nucleic Acids Res. 2022;50(D1):D571–7.

23. Saier MH, Reddy VS, Moreno-Hagelsieb G, Hendargo KJ, Zhang Y, Iddamsetty V, et al. The Transporter Classification Database (TCDB): 2021 update. Nucleic Acids Res. 2021;49(D1):D461–7.

24. The Gene Ontology Consortium. The Gene Ontology knowledgebase in 2023. Genetics. 2023;224(1):iyad031.

25. Chen EZ, Li H. A two-part mixed-effects model for analyzing longitudinal microbiome compositional data. Bioinformatics. 2016 Sep 1;32(17):2611–7.

26. Kosmidis I, Firth D. Jeffreys-prior penalty, finiteness and shrinkage in binomial-response generalized linear models. Biometrika. 2021;108(1):71–82.

27. Mansournia MA, Geroldinger A, Greenland S, Heinze G. Separation in Logistic Regression: Causes, Consequences, and Control. Am J Epidemiol. 2018;187(4):864–70.

28. Dixon P. VEGAN, a package of R functions for community ecology. Journal of Vegetation Science. 2003;14(6):927–30.

29. Stasinopoulos DM, Rigby RA. Generalized additive models for location scale and shape (GAMLSS) in R. Journal of Statistical Software. 2007;23(7):1–46.

**
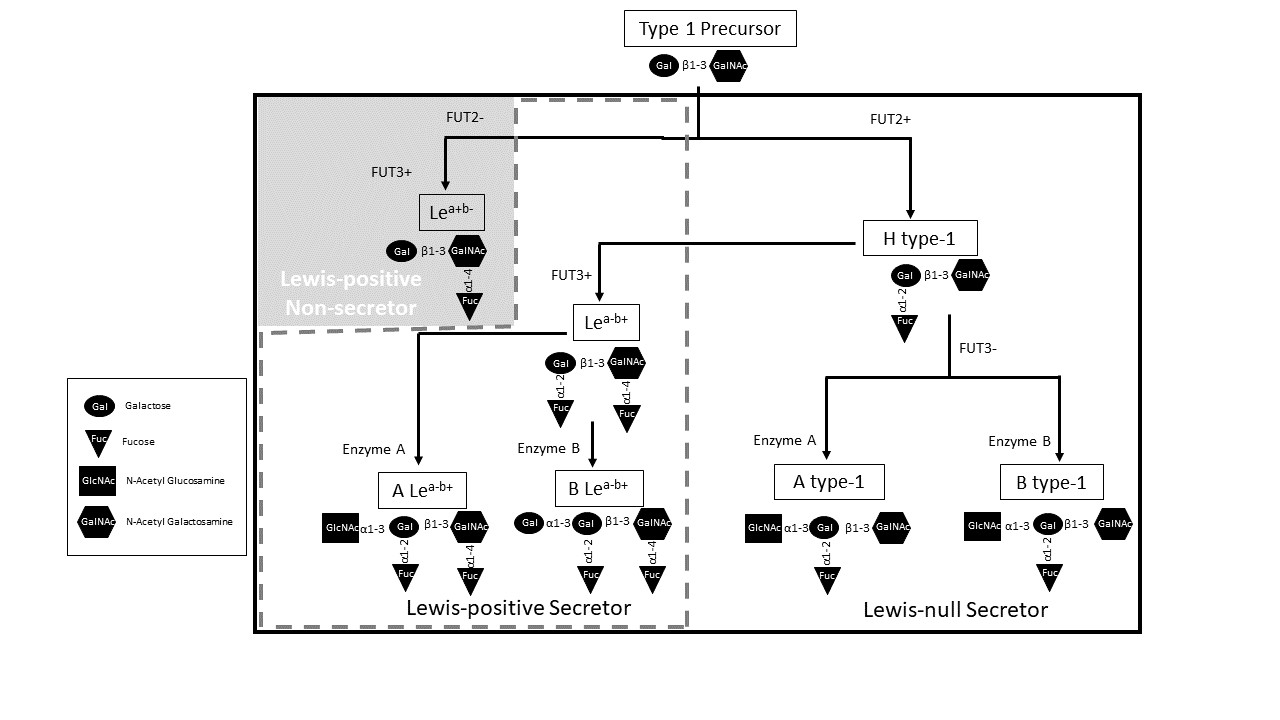
**

**Figure S1. Flow chart of pathways for histo-blood group antigen synthesis by functional FUT2 and FUT3 status.** The type 2 precursor includes β1-4 rather than β1-3 bonds and α1-3 rather than α1-4 bonds. The light-gray shaded region of the chart indicates non-secretor (FUT2-), Lewis-positive (FUT3+) pathways that produce the FUT2-/FUT3+ phenotype (Lewis Le^a^). Pathways outside the light-gray shaded region of the chart produce secretor (FUT2+) phenotypes. In this outside region, the area enclosed by gray dashed lines indicates pathways that produce the FUT2+/FUT3+ (Lewis Le^b^), while the area outside the gray dashed lines indicates the pathways that produce the FUT2+/FUT3- phenotype (Le^a-b-^).

**Figure S2. Flow of participants through the SHINE trial and the analyses reported in this study.** The Sanitation Hygiene Infant Nutrition Efficacy (SHINE) trial enrolled 5280 pregnant women, of whom 3937 were HIV-negative. Of these HIV-negative pregnant women, 1153 and their 1169 live-born infants were recruited into a sub-study to investigate biomarkers of environmental enteric dysfunction. FUT2 and FUT3 phenotypes were ascertained in 792 of these infants and their mothers, of which 610 had complete covariate data for inclusion in regression analyses to determine effect modification of the IYCF intervention my mother-infant FUT2/FUT3 phenotypes. 172 of these infants provided at least 1 fecal specimen for shotgun metagenomic sequencing (354 fecal specimens), of which 348 specimens from 170 infants with complete covariate data were included in longitudinal principal coordinates analyses. 218 of these fecal metagenomes, from 136 infants, plus an additional 66 fecal metagenomes from 43 infants in whom mother and infant FUT2 and FUT3 phenotypes were not ascertained, had sufficient coverage of the *Bifidobacterium longum* genome for pangenome analysis by PanPhlan3.0. 53 infants had metagenomic data at the 6mo follow-up visit and were included in regression analyses to determine effect modification of the IYCF intervention by the infant microbiome.


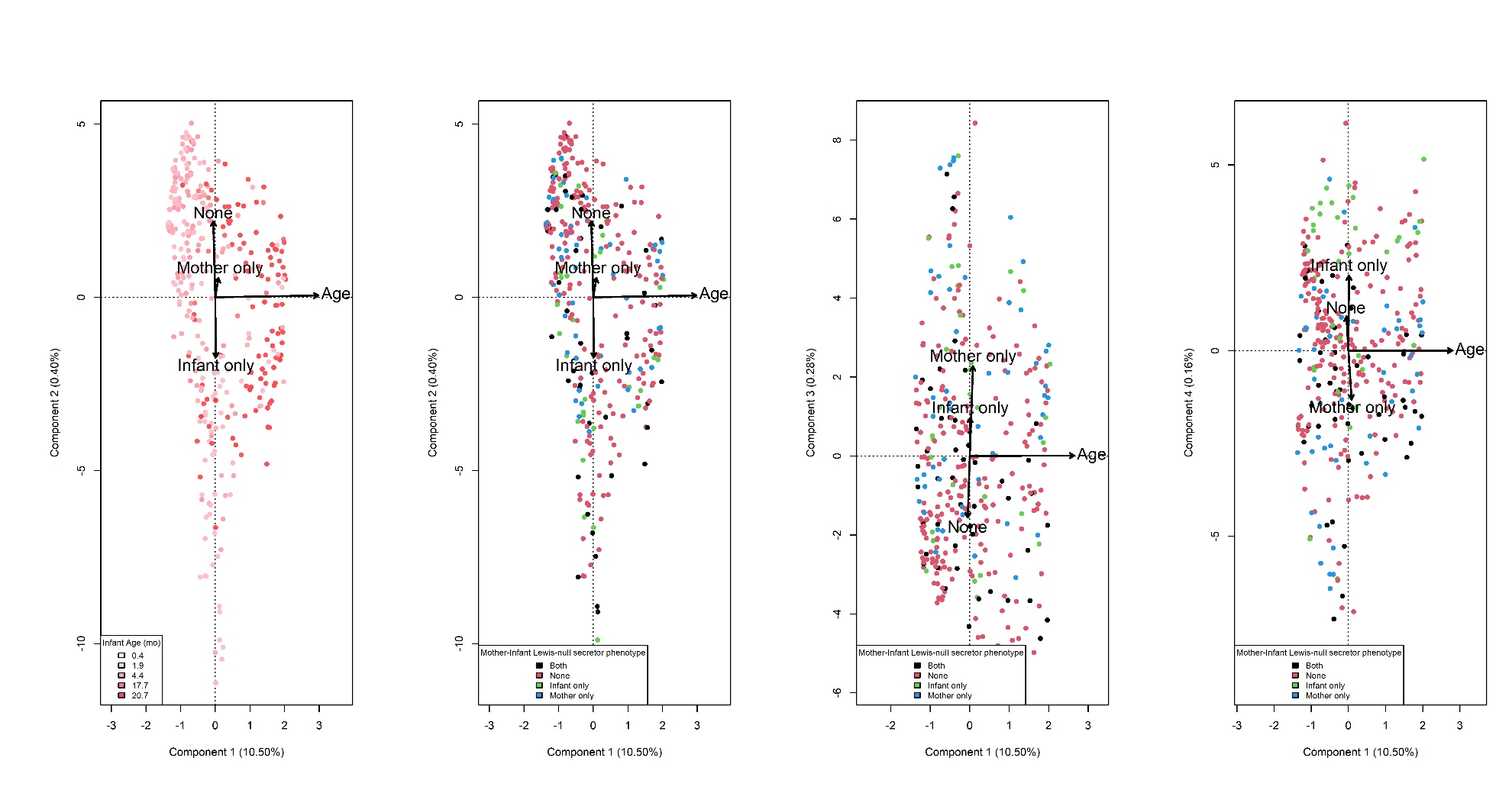


**Figure S3. Infant age and mother-infant FUT2+/FUT3- phenotype significantly explain variation in infant gut microbiome composition.** Ordination biplots for a multivariable constrained PCoA model that included both infant age and mother-infant FUT2+/FUT3- phenotype (N=348 metagenomes from 170 infants collected from 1-18mo with complete covariate data). The x-axis represents PCoA axis 1 scores in all panels. (**a**) The y-axis represents PCoA axis 2, and points are colored from light pink to red to indicate younger to older infant age (mo) at which fecal specimens were taken. (**b-c**) The y-axes represent PCoA axes 2-4, and points are colored to indicate mother-infant FUT2+/FUT3- phenotype concordance, (black, *both* mother and infant are FUT2+/FUT3-; red, *none* are FUT2+/FUT3-; green, *infant only* is a FUT2+/FUT3-; blue, *mother only* is a FUT2+/FUT3-). The direction of embedded arrows indicates the degree of correlation between the corresponding variable and each PCoA axis. Arrows parallel to a PCoA axis are perfectly correlated with that axis’ scores, indicating that variance in microbiome composition explained by the variable indicated are captured by that axis. Arrow length is proportional to the percent variance explained by the variable indicated.

**
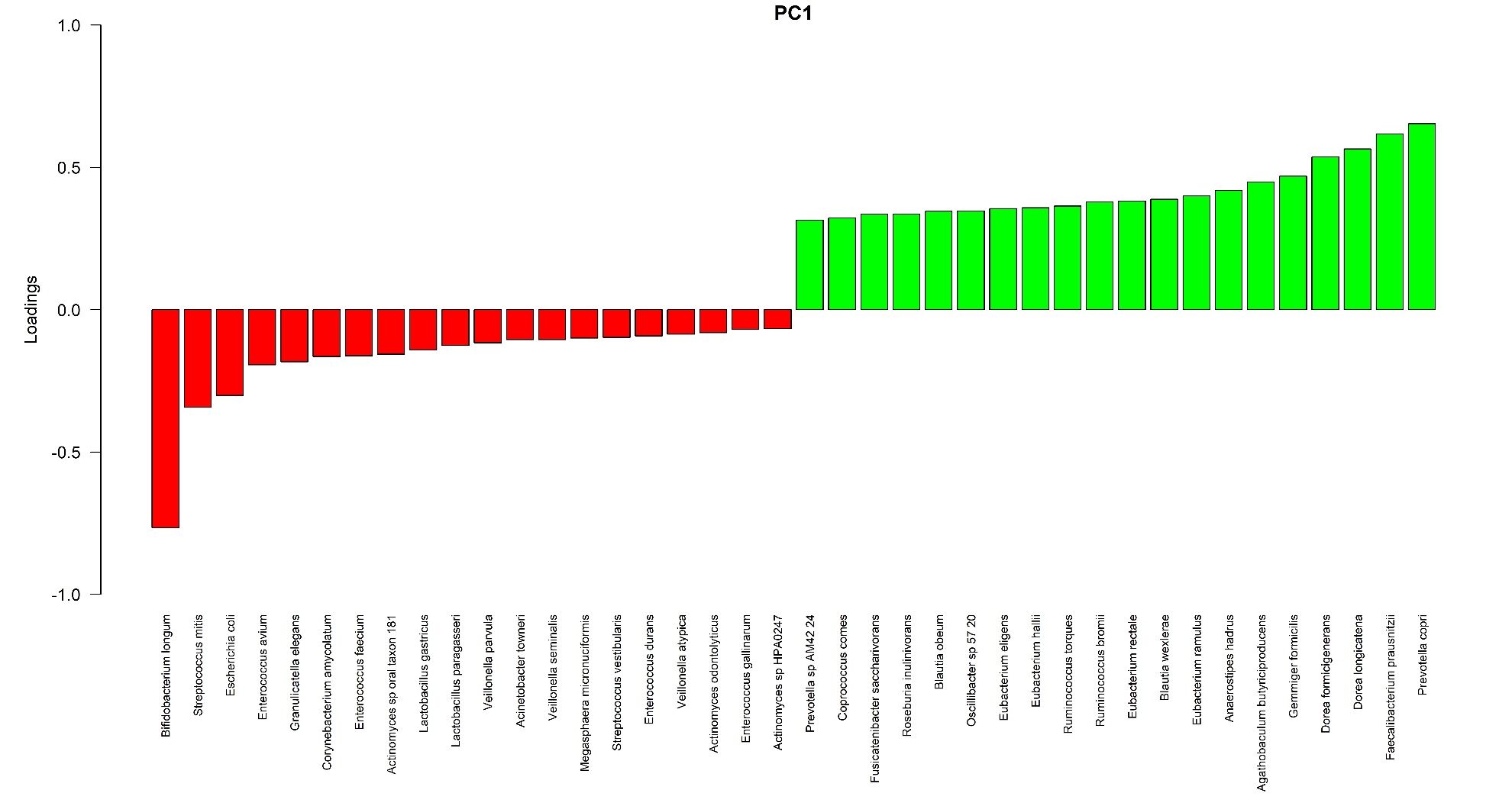
**

**Figure S4. Infant age-related microbiome species turnover predominantly reflects microbiota maturation, characterized by decreased relative abundance of *Bifidobacterium longum* and increased abundance of *Prevotella copri*, *Faecalibacterium prausnitzii*, *Dorea longicatena*, and *Dorea formicigenerans*.** Barplot of microbiome species (x-axis) versus the 20 highest and 20 lowest PCoA axis 1 loadings (y-axis) from a multivariable constrained PCoA model that included both infant age and mother-infant FUT2+/FUT3- phenotype (N=354 metagenomes from 172 infants). Green bars indicate species positively loaded on PCoA axis 1 that are more abundant with increasing infant age, and red bars indicate species negatively loaded on PCoA axis 1 that are less abundant with increasing infant age.


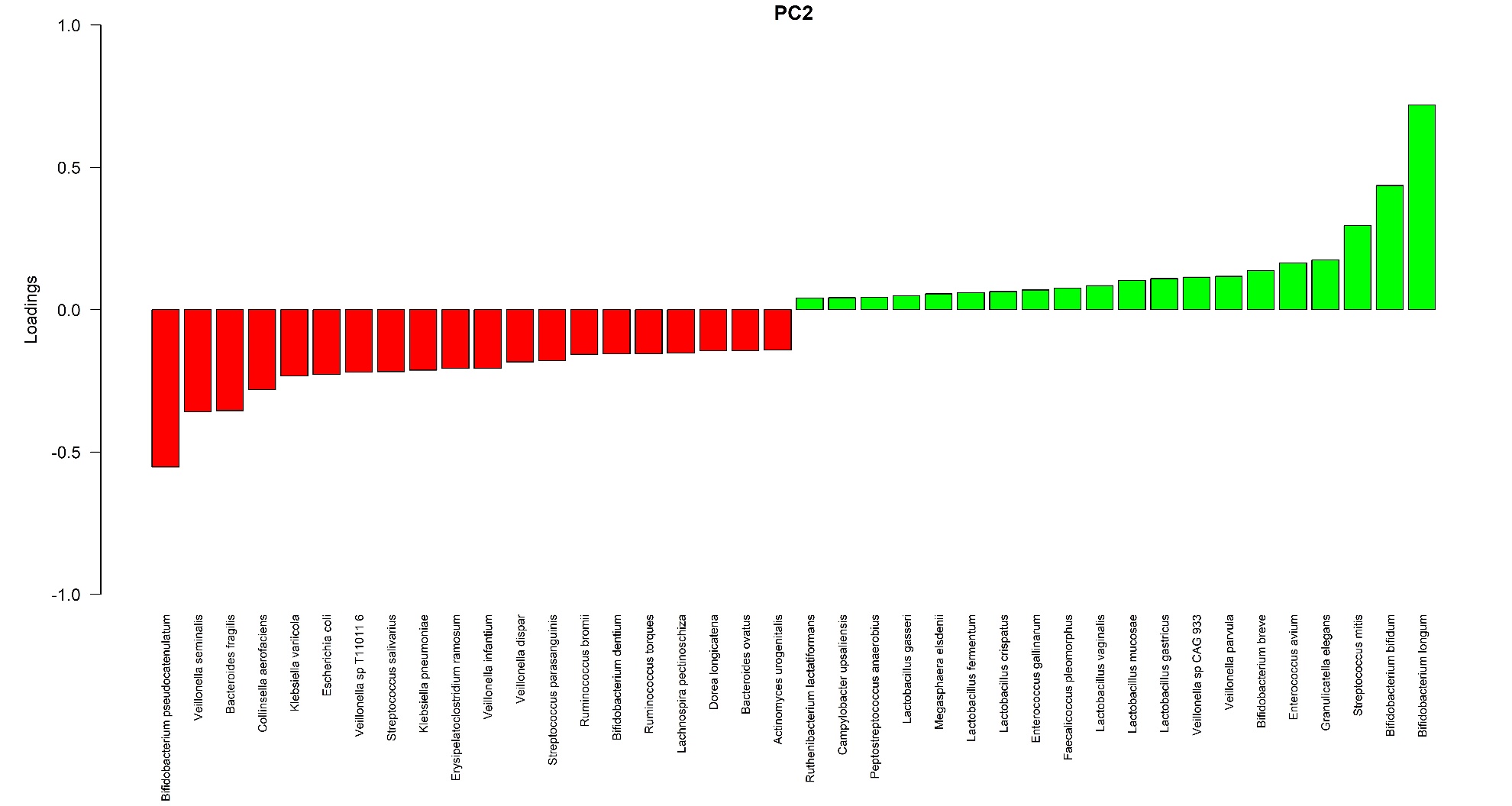


**Figure S5. Mother-infant FUT2+/FUT3- phenotype-related shifts in microbiome species composition predominantly reflects increased relative abundance of *Bifidobacterium longum*.** Barplot of microbiome species (x-axis) versus the 20 highest and 20 lowest PCoA axis 2 loadings (y-axis) from a multivariable constrained PCoA model that included both infant age and mother-infant FUT2+/FUT3- phenotype concordance (N=354 metagenomes from 172 infants). Green bars indicate species positively loaded on PCoA axis 2 that, relative to the *both* group, are more abundant in infants in the *none* FUT2+/FUT3- group and red bars indicate species negatively loaded on PCoA axis 2 that, relative to the *both* group, are more abundant in infants in the *infant only* FUT2+/FUT3- group.


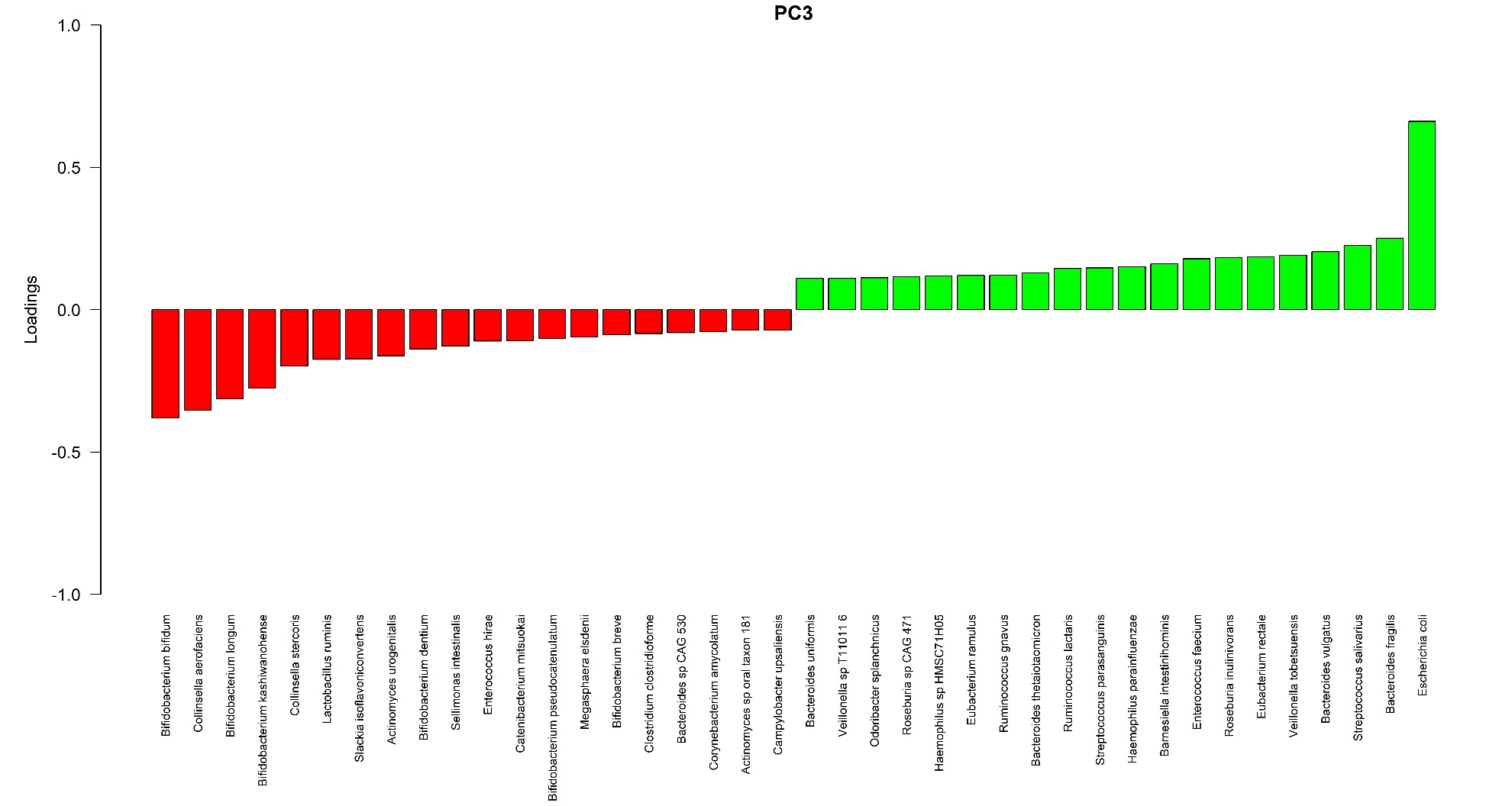


**Figure S6. Mother-infant FUT2+/FUT3- phenotype-related microbiome changes in species composition.** Barplot of microbiome species (x-axis) versus the 20 highest and 20 lowest PCoA axis 3 loadings (y-axis) from multivariable constrained PCoA model that included both infant age and mother-infant FUT2+/FUT3- phenotype concordance (N=354 metagenomes from 172 infants). Green bars indicate species positively loaded on PCoA axis 3 that, relative to the *both* group, are more abundant in infants the *mother only* FUT2+/FUT3- group, and red bars indicate species negatively loaded on PCoA axis 3 that, relative to the *both* group, are more abundant in infants in the *none* FUT2+/FUT3- group.


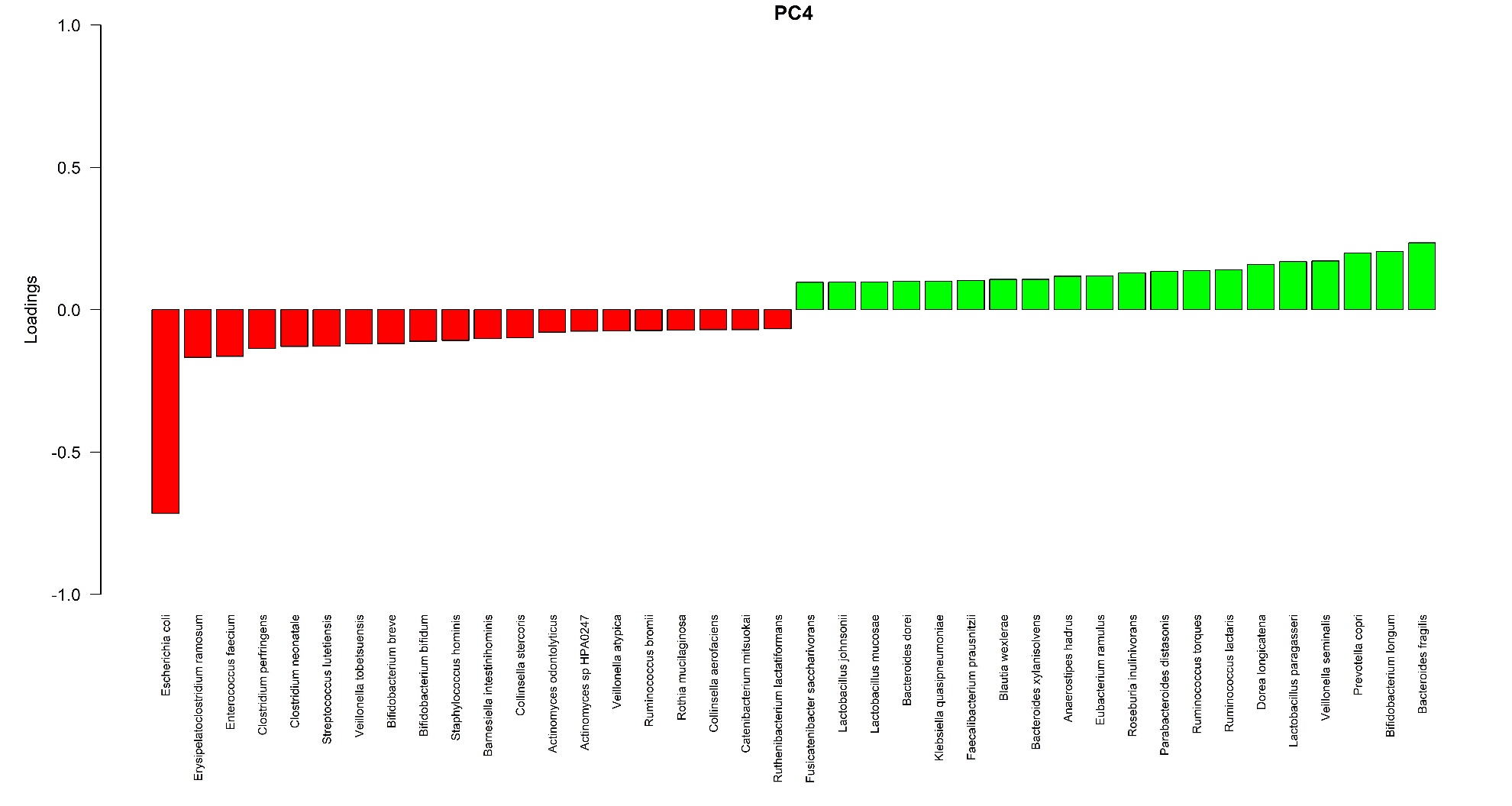


**Figure S7. Mother-infant FUT2+/FUT3- phentoype-related microbiome changes in species composition.** Barplot of microbiome species (x-axis) versus the 20 highest and 20 lowest PCoA axis 4 loadings (y-axis) from a multivariable constrained PCoA model that included both infant age and mother-infant FUT2+/FUT3- phenotype concordance (N=354 metagenomes from 172 infants). Green bars indicate species positively loaded on PCoA axis 4 that, relative to the *both* group, are more abundant in infants in the *infant only* FUT2+/FUT3- group, and red bars indicate species negatively loaded on PCoA axis 4 that, relative to the *both* group, are more abundant in infants in the *mother only* FUT2+/FUT3- group.


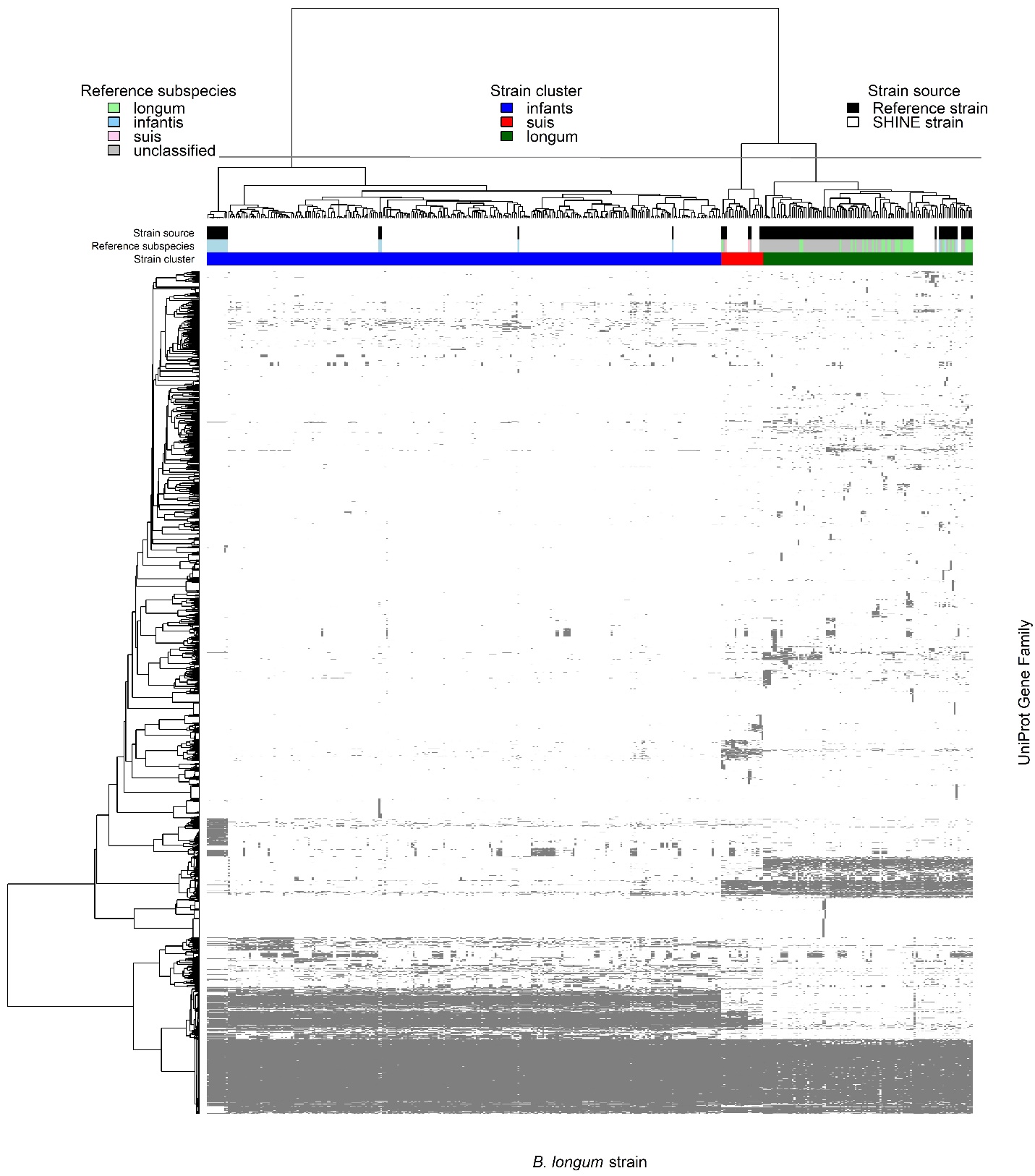


**Figure S8.** **Heatmap of UniProt gene family presence in *Bifidobacterium longum* strains.** UniProt gene family presence is indicated in grey. The horizontal bars at the top from topmost to bottommost row indicate: strain source (black, reference strain; white, SHINE strain); reference strain subspecies (light blue, *B. infantis*; light green, *B. longum longum;* pink, *B. suis*; grey, *unclassified*), and strain cluster (blue, *B. infantis*; dark green, *B. longum longum*; red, *B. suis*). Clusters were defined by hierarchical clustering of Jaccard dissimilarities, and cluster membership was determined by cutting the cluster dendrogram at a height that would produce three clusters (horizontal gray line). Rows are ordered using the default hierarchical clustering settings of *heatmap3*. (N=284 SHINE strains and 118 reference strains).


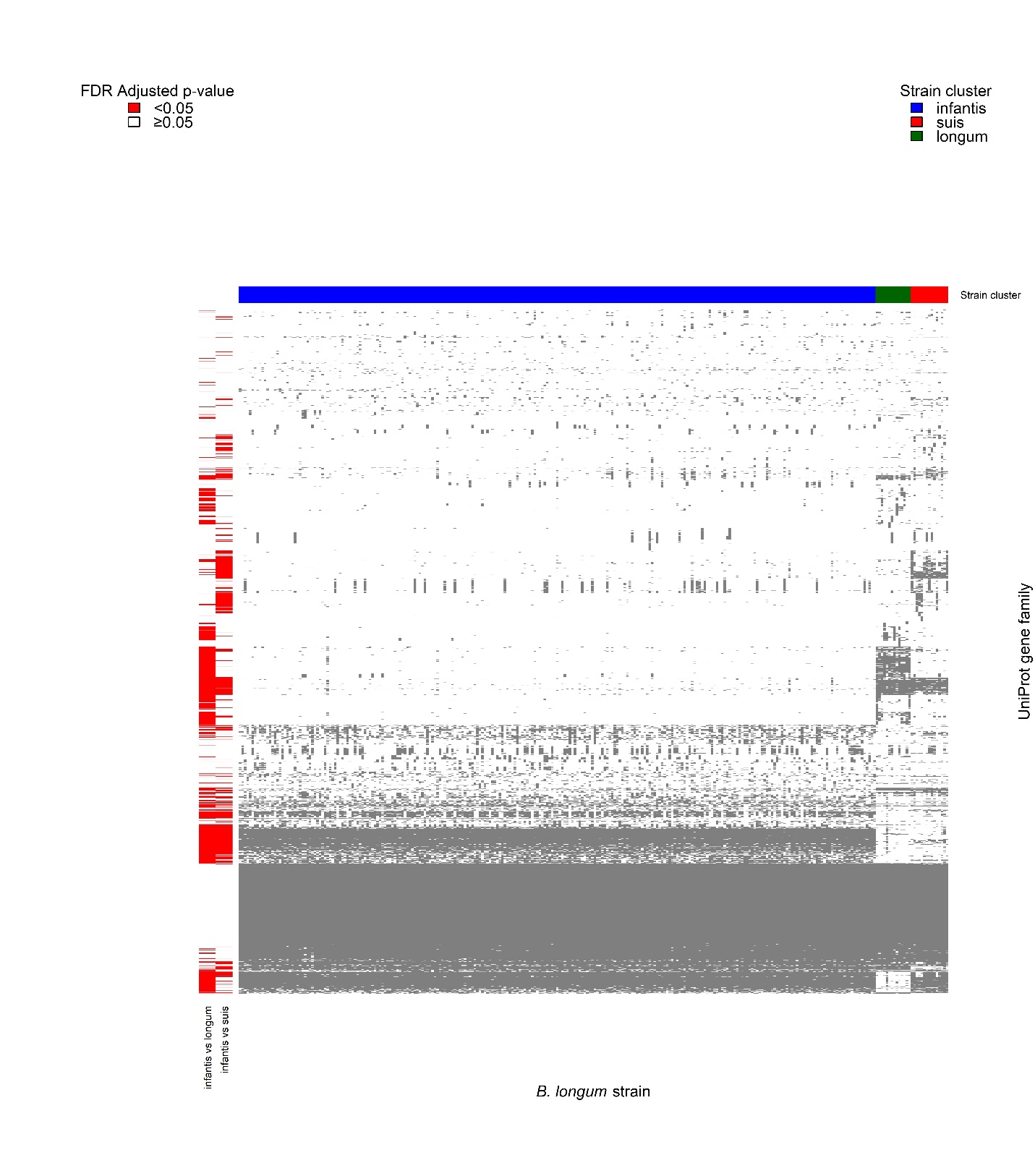


**Figure S9. Differences in UniProt gene family profiles between SHINE infant *Bifidobacterium longum* strain clusters.** Heatmap of UniProt gene family presence in SHINE infant *B. longum* strains (N=284). UniProt gene family presence is indicated in grey. The horizontal bar at the top indicates strain cluster (blue, *B. infantis*; dark green, *B. longum longum*; red, *B. suis*). Vertical bars from left to right indicate: UniProt gene families that differed in prevalence between the *B. infantis* and *B. longum longum* cluster by two-sided Fisher’s Exact test after FDR correction (red); UniProt gene families that differed in prevalence between the *B. infantis* and *B. suis* cluster by two-sided Fisher’s Exact test after FDR correction (red).


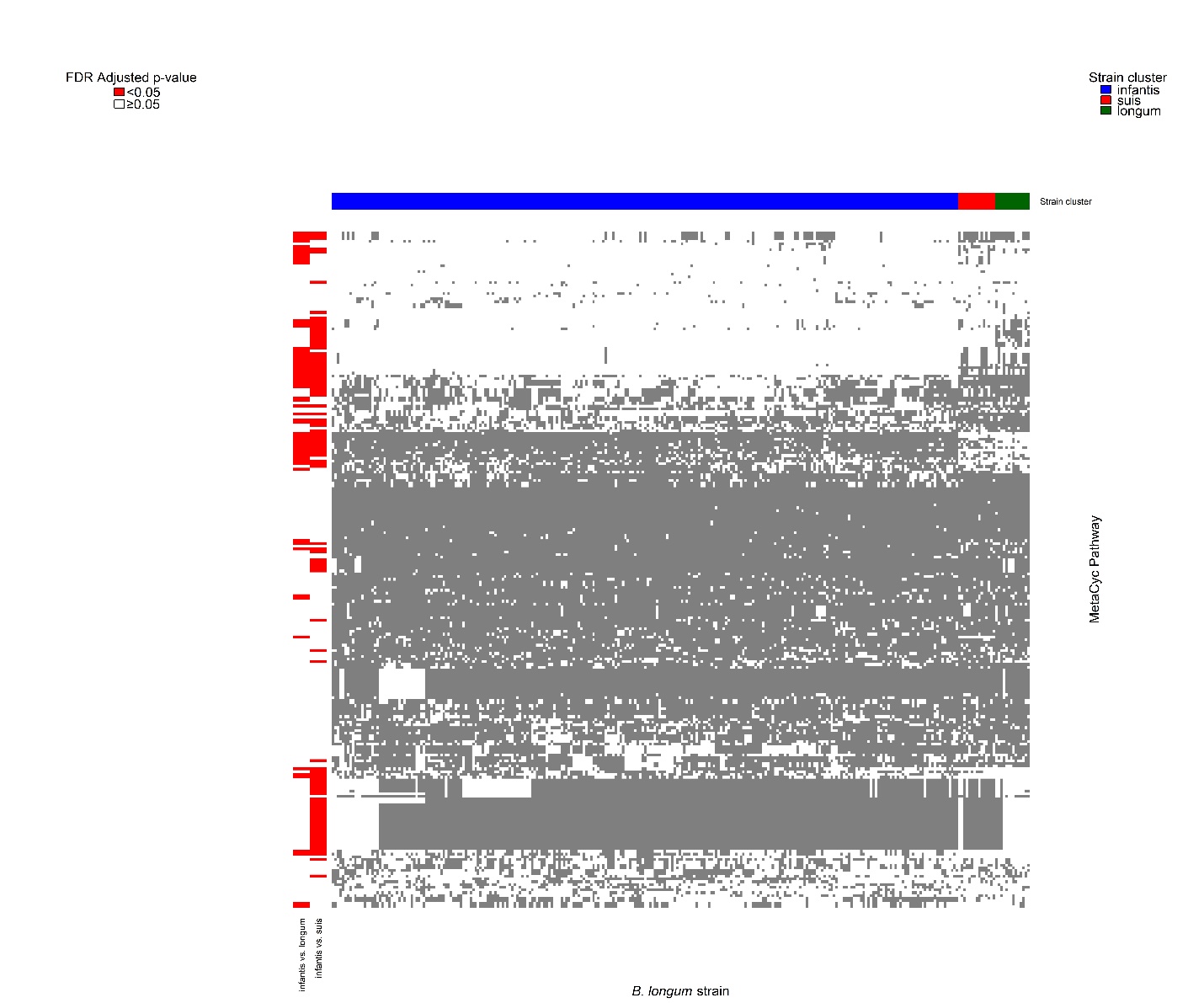


**Figure S10. Differences in MetaCyc pathway profiles between SHINE infant *Bifidobacterium longum* strain clusters.** Heatmap of MetaCyc pathway presence in SHINE infant *B. longum* strains (N=284) as determined by MinPath. MetaCyc pathway presence is indicated in grey. The horizontal bar at the top indicates strain cluster (blue, *B. infantis*; dark green, *B. longum longum*; red, *B. suis*). Vertical bars from left to right indicate: MetaCyc pathways that differed in prevalence between the *B. infantis* and *B. longum longum* cluster by two-sided Fisher’s Exact test after FDR correction (red); MetaCyc pathways that differed in prevalence between the *B. infantis* and *B. suis* cluster by two-sided Fisher’s Exact test after FDR correction (red).

**
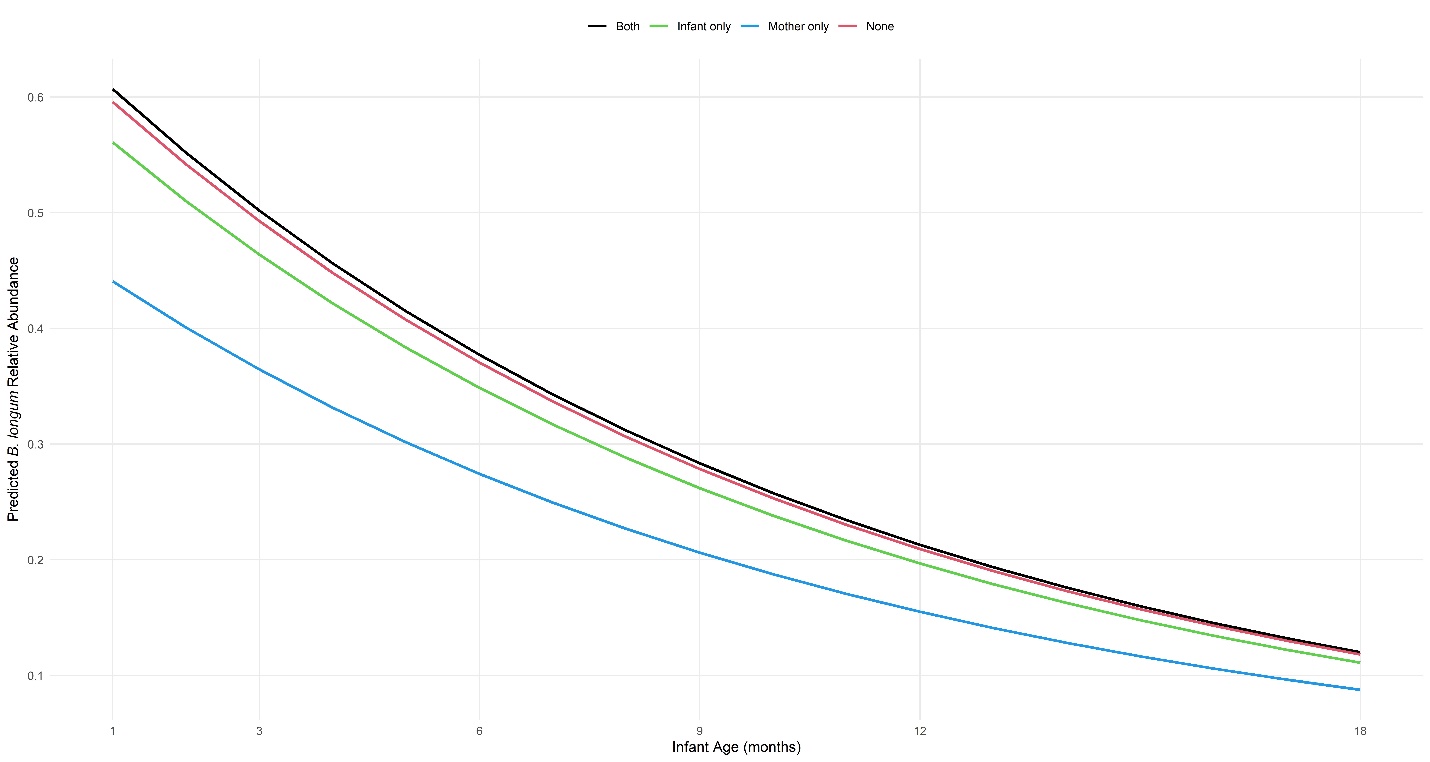
**

**Figure S11.** **Relative abundance of *Bifidobacterium longum* in the infant gut microbiome over time.** Multivariable zero-inflated mixed-effects model fitted to longitudinal data (N=225 specimens from 87 infants). Infant age and mother-infant FUT2+/FUT3- phenotype were significant predictors of *B. longum* relative abundance. The model was used to predict the mean *B. longum* relative abundance (y-axis) which was plotted against infant age (mo) at follow-up (x-axis), conditional on model covariates. Mother-infant FUT2+/FUT3- phenotypes are differentiated by color (black, *both*; red, *none*; green, *infant only*; blue, *mother only* are FUT2+/FUT3-).

**
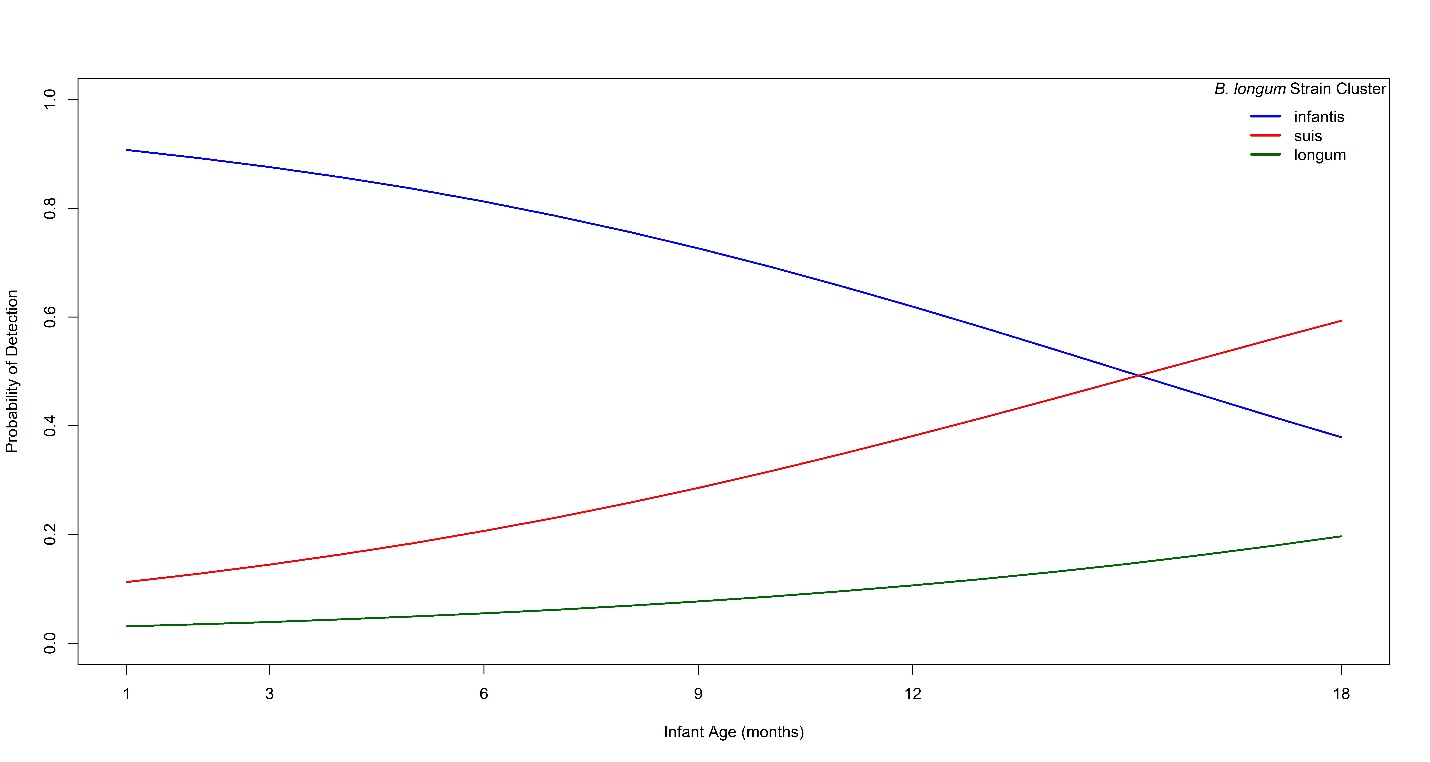
**

**Figure S12.** **Probability of *Bifidobacterium longum* strain cluster detection over time.** Multivariable logistic regression models were fitted to longitudinal data (N=133 specimens from 70 infants) with detection of each strain cluster as the dependent variable. A separate model was fitted for each strain cluster. Each model was used to predict the probability of detecting a *B. longum* strain in the specified cluster (y-axis) which was plotted against infant age (mo) at follow-up (x-axis), conditional on model covariates. Line colors indicate strain cluster (blue, *B. infantis*; green, *B. longum longum*; red, *B. suis*). *B. infantis* cluster strains were most likely to be detected throughout follow-up, but decreased in detection probability over time and was lowest at 18mo of age. While the probability of detecting *B. longum longum* and *B. suis* cluster strains increased with infant age. *B. suis* cluster strain detection probabilities increased with age more than *B. longum longum* cluster strain detection and peaked at 18mo of age, where it was more likely to be detected than strains in the *B. infantis* cluster.

**
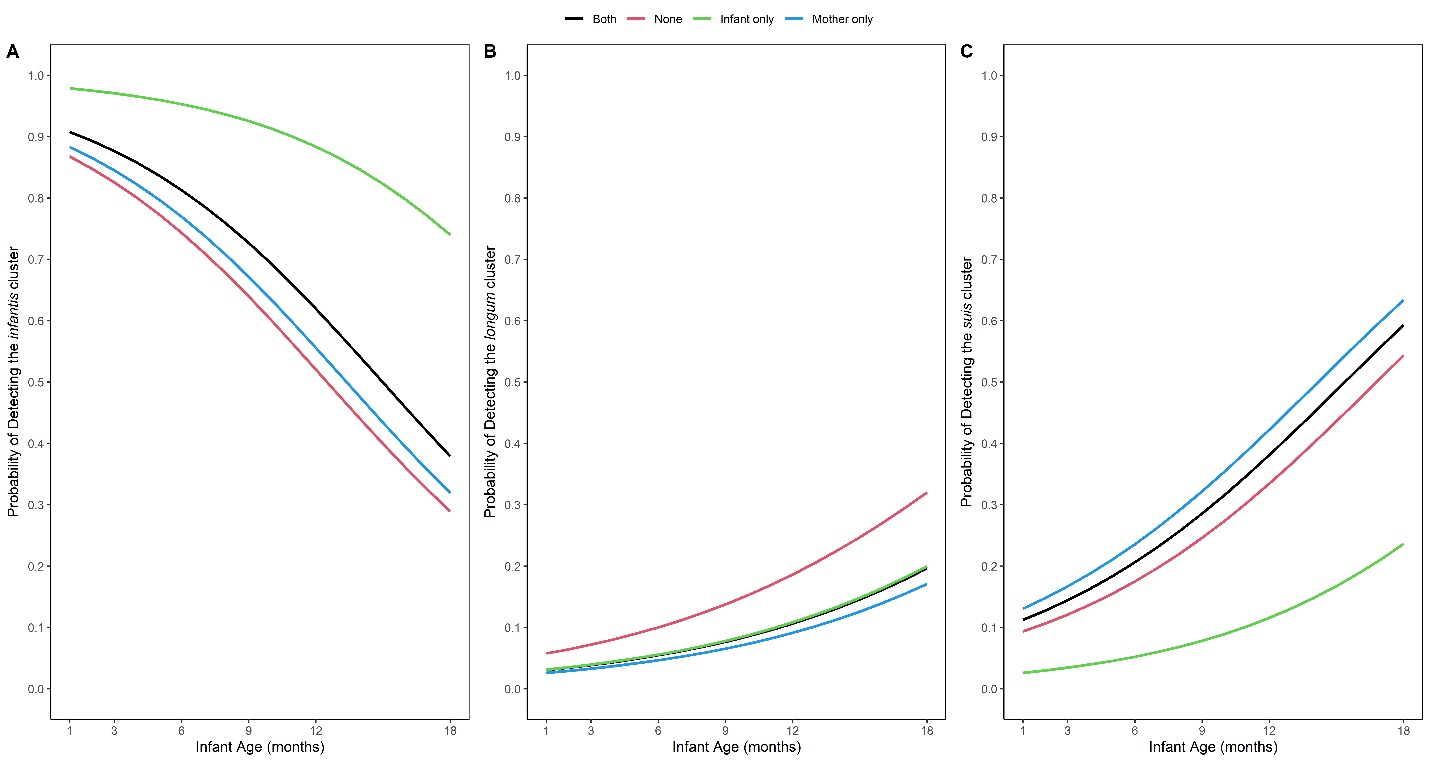
**

**Figure S13. Probability of *Bifidobacterium longum* strain cluster detection over time by mother-infant FUT2+/FUT3- phenotype.** Multivariable logistic regression models were fitted to longitudinal data (N=133 specimens from 70 infants) with detection of each strain cluster as the dependent variable. A separate model was fitted for each strain cluster. Each model was used to predict the probability of detecting a *B. longum* strain in the specified cluster (y-axis) which was plotted against infant age (mo) at follow-up (x-axis) and mother-infant FUT2+/FUT3- phenotype, conditional on model covariates. Mother-infant FUT2+/FUT3- phenotypes are differentiated by color (black, *both*; red, *none*; green, *infant only*; blue, *mother only* are FUT2+/FUT3-). The *infant only* group had the highest probability of the *B. infantis* cluster throughout follow-up. While the *mother only* group had a lower probability of carrying an *B. infantis* cluster strain. The *mother only* group also had the highest probability of carrying a *B. suis* cluster strain. (**a**) *B. infantis* cluster strain detection. (**b**) *B. longum longum* cluster strain detection. (**c**) *B. suis* cluster strain detection.

| **Table S1. Baseline characteristics of infants in this substudy are comparable to the wider SHINE trial cohort** | | | |
| --- | --- | --- | --- |
| **Variable** | **Microbiome Substudy** | **6mo Visit** | **Not enrolled in the microbiome substudy** |
| N | 236 | 53 | 2784 |
| Trial arm |  |  |  |
| IYCF | 119(50.4%) | 18(34.0%) | 1413(50.1%) |
| non-IYCF | 117(49.6%) | 35(66.0%) | 1407(49.9%) |
| WASH | 108(45.8%) | 25(47.2%) | 1564(55.5%) |
| non-WASH | 128(54.2%) | 28(52.8%) | 1256(44.5%) |
| Household size |  |  |  |
| Median[Q1,Q3] | 5.00[4.00,6.00] | 5.00[4.00,6.00] | 5.00[3.00,6.00] |
| Wealth quintile |  |  |  |
| 1 (lowest) | 33(14.2%) | 9(17.0%) | 477(18.7%) |
| 2 | 51(22.0%) | 14(26.4%) | 484(19.0%) |
| 3 | 49(21.1%) | 9(17.0%) | 507(19.9%) |
| 4 | 58(25.0%) | 17(32.1%) | 548(21.5%) |
| 5 (highest) | 41(17.7%) | 4(7.5%) | 534(20.9%) |
| Mother age, years |  |  |  |
| Mean (sd) | 27.3(6.2) | 28.1(6.9) | 25.3(6.5) |
| Mother height, cm |  |  |  |
| Mean (sd) | 160.4(5.8) | 158.8(5.8) | 159.7(8.9) |
| Mother mid-upper arm circumference, cm |  |  |  |
| Mean (sd) | 27.2(3.4) | 26.7(3.8) | 26.3(3.0) |
| Mother years of schooling completed |  |  |  |
| Mean (sd) | 10.5(1.8) | 10.8(1.3) | 9.6(1.8) |
| Mother marital status |  |  |  |
| Yes | 217(96.0%) | 46(90.2%) | 2491(95.7%) |
| Mother employed |  |  |  |
| Yes | 23(9.9%) | 3(5.7%) | 311 (8.5%) |
| Mother religion |  |  |  |
| Apostolic | 94(41.0%) | 22(43.1%) | 1223(46.6%) |
| Other Christian | 126(55.0%) | 25(49.0%) | 217(8.3%) |
| Other | 9(3.9%) | 4(7.8%) | 220(8.4%) |
| Mother potential depression (≥ 10 on Edinburgh Postnatal Depression Scale) |  |  |  |
| Yes | 15(6.6%) | 4(7.7%) | 264(10.8%) |
| Parity |  |  |  |
| Median[Q1,Q3] | 2.00[1.00,3.00] | 2.00[1.00,3.00] | 2.00[1.00,3.00] |
| Ever booked antenatal care |  |  |  |
| Yes | 217(99.5%) | 47(97.9%) | 2624(99.2%) |
| Household meets minimum Diet Diversity Score |  |  |  |
| yes | 102(49.3%) | 25(53.2%) | 827(37.0%) |
| Coping Strategies Index score |  |  |  |
| Median[Q1,Q3] | 0.00[0.00,3.00] | 0.00[0.00,3.50] | 1.00[0.00,8.00] |
| Female Sex |  |  |  |
| Yes | 101(42.8%) | 28(52.8%) | 1395(49.7%) |
| Birthweight, kg |  |  |  |
| Mean (sd) | 3.1(0.5) | 3.1(0.5) | 3.1(0.5) |
| Low Birthweight (<2500 g) |  |  |  |
| Yes | 16(6.9%) | 5(9.6%) | 236(9.6%) |
| Preterm (<37 weeks gestation) |  |  |  |
| Yes | 21(13.0%) | 9(20.9%) | 250(17.8%) |
| Ever exclusively breastfed at 3mo |  |  |  |
| Yes | 92(82.9%) | 31(81.6%) | 3026(88.7%) |
| Vaginal delivery |  |  |  |
| Yes | 216(91.5%) | 45(84.9%) | 2335(92.4%) |
| Institutional delivery |  |  |  |
| Yes | 212(91.8%) | 48(90.6%) | 2202(88.7%) |
| Delivery occurred during hungry season |  |  |  |
| Yes | 74(31.4%) | 13(24.5%) | 1583(56.2%) |
| Stunted at 18mo |  |  |  |
| Yes | 61(26.9%) | 17(32.1%) | 1134(30.9%) |
| LAZ at 18mo |  |  |  |
| Mean (sd) | -1.4(1.1) | -1.6(1.0) | -1.5(1.1) |
| Mother-infant Lewis Ag phenotype concordance |  |  |  |
| Both FUT2+/FUT3- | 26(15.1%) | 7(13.2%) | N/A |
| None FUT2+/FUT3- | 108(62.8%) | 31(58.5%) | N/A |
| Infant only FUT2+/FUT3- | 15(8.7%) | 5(9.4%) | N/A |
| Mother only FUT2+/FUT3- | 23(13.4%) | 10(18.9%) | N/A |
|  |  |  |  |

| **Table S2. Distribution of mother and infant FUT2 and FUT3 status and combinations** | | | | | | | | |  |
| --- | --- | --- | --- | --- | --- | --- | --- | --- | --- |
|  |  | **Infant’s status** | | | | **Mother’s status** | | | |
|  |  | **FUT2+** | **FUT2-** | **FUT3+** | **FUT3-** | **FUT2+** | **FUT2-** | **FUT3+** | **FUT3-** |
| **Infant’s status** | **FUT3+** | 635 (64.9%) | 117 (11.9%) |  |  |  |  |  |  |
|  | **FUT3-** | 227 (23.2%) | 0 (0.0%) |  |  |  |  |  |  |
| **Mother’s status** | **FUT2+** | 629 (79.4%) | 60 (7.5%) | 524 (66.2%) | 165 (20.8%) |  |  |  |  |
|  | **FUT2-** | 71 (8.9%) | 32 (4.0%) | 92 (11.6%) | 11 (1.4%) |  |  |  |  |
|  | **FUT3+** | 517 (65.3%) | 80 (10.1%) | 509 (64.3%) | 88 (11.1%) | 538 (60.5%) | 128 (14.4%) |  |  |
|  | **FUT3-** | 183 (23.1%) | 12 (1.5%) | 107 (13.5%) | 88 (11.1%) | 223 (25.1%) | 0 (0.0%) |  |  |
| **Total** |  | 862 (88.1%) | 117 (11.9%) | 752 (76.8%) | 227 (23.2%) | 761 (85.6%) | 128 (14.4%) | 666 (74.9%) | 223 (25.1%) |
| **Indeterminate** |  | 125 | | 125 | | 110 | | 110 | |
| **Not Tested** |  | 65 | | 65 | | 170 | | 170 | |
|  |  |  |  |  |  |  |  |  |  |

**Table S3. Distribution of paired mother and infant FUT2 and FUT3 phenotype combinations**

|  |  | **Mother FUT2+/FUT3-** | |
| --- | --- | --- | --- |
|  |  | **Yes** | **No** |
| Infant FUT2+/FUT3- | Yes | 88 (11.1%) | 88 (11.1%) |
|  | No | 107 (13.5%) | 509 (64.3%) |
|  |  |  |  |
|  |  |  |  |
|  |  | **Mother FUT2+/FUT3+** | |
|  |  | **Yes** | **No** |
| Infant FUT2+/FUT3+ | Yes | 369 (46.6%) | 155 (19.6%) |
|  | No | 125 (15.8%) | 143 (18.1%) |
|  |  |  |  |
|  |  |  |  |
|  |  | **Mother FUT2-/FUT3+** | |
|  |  | **Yes** | **No** |
| Infant FUT2-/FUT3+ | Yes | 32 (4.0%) | 60 (7.6%) |
|  | No | 71 (9.0%) | 629 (79.4%) |
|  |  |  |  |

**Table S4. Number stunted / Total N per subgroup by IYCF and mother-infant FUT2 and FUT3 phenotype combinations used in the multivariable regression models to determine modification of the IYCF intervention effect on stunting at 18mo by mother-infant FUT2 and FUT3 phenotypes which are presented in Table 1 and Table S5.**

|  | No-IYCF | IYCF |
| --- | --- | --- |
| Both FUT2-/FUT3+ | 2/7 | 3/15 |
| None FUT2-/FUT3+ | 67/224 | 60/260 |
| Infant only FUT2-/FUT3+ | 4/23 | 6/24 |
| Mother only FUT2-/FUT3+ | 4/24 | 8/33 |
|  |  |  |
| Both FUT2+/FUT3+ | 39/125 | 33/151 |
| None FUT2+/FUT3+ | 13/54 | 19/55 |
| Infant only FUT2+/FUT3+ | 16/52 | 16/74 |
| Mother only FUT2+/FUT3+ | 9/47 | 9/52 |
|  |  |  |
| Both FUT2+/FUT3- | 7/38 | 10/31 |
| None FUT2+/FUT3- | 45/170 | 44/214 |
| Infant only FUT2+/FUT3- | 9/33 | 9/37 |
| Mom only FUT2+/FUT3- | 16/37 | 14/50 |

**Table S5. Multivariable regression model^1^ to estimate modification of IYCF on stunting at 18mo by mother-infant FUT2 and FUT3 phenotype combinations among 610 infants in whom mother and infant FUT2 and FUT3 status was ascertained and complete covariate data were available**

|  | **Main Effects** | | | **IYCF-by-FUT2 and FUT3 phenotype combination interaction effects** | | |
| --- | --- | --- | --- | --- | --- | --- |
|  | **PD (95%CI)** | **p-value** | **Adjusted p-value^2^** | **Prevalence difference-in-differences (95%CI)** | **p-value** | **Adjusted p-value^2^** |
| Both FUT2-/FUT3+ | ref | ref | ref | ref | ref | ref |
| None FUT2-/FUT3+ | -9.3%(-38.2%,19.7%) | 0.531 | 0.531 | 17.5%(-14.7%,49.7%) | 0.286 | 0.286 |
| Infant only FUT2-/FUT3+ | -18.4%(-49.8%,13.0%) | 0.250 | 0.374 | 30.8%(-6.4%,68.0%) | 0.104 | 0.191 |
| Mother only FUT2-/FUT3+ | -17.0%(-49.8%,15.9%) | 0.312 | 0.312 | 23.3%(-15.0%,61.5%) | 0.233 | 0.350 |
| IYCF | -22.3%(-54.6%,10.1%) | 0.177 | 0.177 |  |  |  |
|  |  |  |  |  |  |  |
|  |  |  |  |  |  |  |
|  | **Main Effects** | | | **IYCF-by-FUT2 and FUT3 phenotype combination interaction effects** | | |
|  | **PD (95%CI)** | **p-value** | **Adjusted p-value^2^** | **Prevalence difference-in-differences (95%CI)** | **p-value** | **Adjusted p-value^2^** |
| Both FUT2+/FUT3+ | ref | ref | ref | ref | ref | ref |
| None FUT2+/FUT3+ | -7.8%(-20.3%,4.7%) | 0.221 | 0.331 | 14.6%(-3.4%,32.5%) | 0.112 | 0.168 |
| Infant only FUT2+/FUT3+ | 2.1%(-10.3%,14.4%) | 0.741 | 0.741 | -4.1%(-18.9%,10.6%) | 0.582 | 0.582 |
| Mother only FUT2+/FUT3+ | -8.9%(-21.1%,3.3%) | 0.151 | 0.227 | 6.7%(-9.1%,22.4%) | 0.406 | 0.406 |
| IYCF | -6.8%(-15.5%,1.9%) | 0.128 | 0.177 |  |  |  |

^1^Covariates include season of birth, birthweight, infant sex, ever exclusive breastfeeding at 3mo, age at the 6mo visit, infant WHZ at the 6mo visit, and infant LAZ at the 6mo visit. N’s are reported in Supplemental Table S4.

^2^Adjusted for multiple hypothesis testing by the Benjamini-Hochberg method

PD, prevalence difference; 95%CI, 95% confidence interval; ref, referent; IYCF, infant and you child feeding

**Table S6. Multivariable regression model^1^ to estimate modification of IYCF on LAZ at 18mo by mother-infant FUT2 and FUT3 phenotype combinations among 610 infants in whom mother and infant FUT2 and FUT3 status was ascertained and complete covariate data were available**

|  | **Main Effects** | | | **IYCF-by-FUT2 and FUT3 phenotype combination interaction effects** | | |
| --- | --- | --- | --- | --- | --- | --- |
|  | **ΔLAZ (95%CI)** | **p-value** | **Adjusted p-value^2^** | **LAZ difference-in-differences (95%CI)** | **p-value** | **Adjusted p-value^2^** |
| Both FUT2-/FUT3+ | ref | ref | ref | ref | ref | ref |
| None FUT2-/FUT3+ | 0.26(-0.28,0.79) | 0.346 | 0.519 | -0.58(-1.20,0.05) | 0.072 | 0.216 |
| Infant only FUT2-/FUT3+ | 0.29(-0.34,0.91) | 0.369 | 0.881 | -0.60(-1.38,0.18) | 0.134 | 0.256 |
| Mother only FUT2-/FUT3+ | 0.54(-0.12,1.20) | 0.110 | 0.177 | -0.88(-1.64,-0.12) | 0.023 | 0.070 |
| IYCF | 0.64(0.01,1.27) | 0.045 | 0.135 |  |  |  |
|  |  |  |  |  |  |  |
|  |  |  |  |  |  |  |
|  | **Main Effects** | | | **IYCF-by-FUT2 and FUT3 phenotype combination interaction effects** | | |
|  | **ΔLAZ (95%CI)** | **p-value** | **Adjusted p-value^2^** | **LAZ difference-in-differences (95%CI)** | **p-value** | **Adjusted p-value^2^** |
| Both FUT2+/FUT3+ | ref | ref | ref | ref | ref | ref |
| None FUT2+/FUT3+ | -0.13(-0.37,0.10) | 0.262 | 0.519 | 0.25(-0.09,0.58) | 0.146 | 0.219 |
| Infant only FUT2+/FUT3+ | -0.02(-0.27,0.23) | 0.869 | 0.881 | 0.22(-0.09,0.53) | 0.171 | 0.256 |
| Mother only FUT2+/FUT3+ | 0.08(-0.17,0.33) | 0.528 | 0.528 | 0.07(-0.28,0.41) | 0.696 | 0.696 |
| IYCF | -0.04(-0.23,0.14) | 0.657 | 0.860 |  |  |  |
|  |  |  |  |  |  |  |
|  |  |  |  |  |  |  |
|  | **Main Effects** | | | **IYCF-by-FUT2 and FUT3 phenotype combination interaction effects** | | |
|  | **ΔLAZ (95%CI)** | **p-value** | **Adjusted p-value^2^** | **LAZ difference-in-differences (95%CI)** | **p-value** | **Adjusted p-value^2^** |
|  | ref | ref | ref | ref | ref | ref |
| Both FUT2+/FUT3- | 0.08(-0.22,0.38) | 0.611 | 0.611 | -0.08(-0.50,0.35) | 0.726 | 0.726 |
| None FUT2+/FUT3- | 0.03(-0.34,0.39) | 0.881 | 0.881 | 0.05(-0.51,0.61) | 0.855 | 0.855 |
| Infant only FUT2+/FUT3- | -0.26(-0.59,0.07) | 0.118 | 0.177 | 0.45(-0.05,0.96) | 0.079 | 0.118 |
| IYCF | 0.04(-0.38,0.45) | 0.860 | 0.860 |  |  |  |

^1^Covariates include season of birth, birthweight, infant sex, ever exclusive breastfeeding at 3mo, age at the 6mo visit, infant WHZ at the 6mo visit, and infant LAZ at the 6mo visit. N’s are reported in Supplemental Table S7

^2^Adjusted for multiple hypothesis testing by the Benjamini-Hochberg method

ΔLAZ, difference in length-for-age z-score; 95%CI, 95% confidence interval; ref, referent; IYCF, infant and you child feeding

**Table S7.** **Total N per subgroup by IYCF and mother-infant FUT2 and FUT3 phenotype combinations used in the multivariable regression models to estimate modification of the IYCF intervention effect on LAZ at 18mo by mother-infant FUT2 and FUT3 phenotypes which are presented in Table S6.**

|  | No-IYCF | IYCF |
| --- | --- | --- |
| Both FUT2-/FUT3+ | 7 | 15 |
| None FUT2-/FUT3+ | 224 | 260 |
| Infant only FUT2-/FUT3+ | 23 | 24 |
| Mother only FUT2-/FUT3+ | 24 | 33 |
|  |  |  |
| Both FUT2+/FUT3+ | 125 | 151 |
| None FUT2+/FUT3+ | 54 | 55 |
| Infant only FUT2+/FUT3+ | 52 | 74 |
| Mother only FUT2+/FUT3+ | 47 | 52 |
|  |  |  |
| Both FUT2+/FUT3- | 38 | 31 |
| None FUT2+/FUT3- | 170 | 214 |
| Infant only FUT2+/FUT3- | 33 | 37 |
| Mom only FUT2+/FUT3- | 37 | 50 |

**Table S8. Multivariable regression models^1^ to estimate modification of IYCF on LAZ at 18mo by infant gut microbiome species turnover in 53 infants**

|  | **N** | **ΔLAZ (95%CI)** | **p-value** | **Adjusted p-value^2^** |
| --- | --- | --- | --- | --- |
| PCoA Axis 1 |  |  | | |
| IYCF | 53 | 0.22(-0.74,1.19) | 0.590 | 0.833 |
| PC1 | 53 | -0.12(-0.72,0.48) | 0.657 | 0.858 |
| PC1-by-IYCF^3^ | 53 | 0.78(-0.46,2.02) | 0.076 | 0.263 |
| PCoA Axis 2 |  |  | | |
| IYCF | 53 | -0.08(-0.69,0.54) | 0.821 | 0.829 |
| PC2 | 53 | 0.02(-0.13,0.17) | 0.821 | 0.829 |
| PC2-by-IYCF^3^ | 53 | -0.18(-0.43,0.08) | 0.100 | 0.263 |
| PCoA Axis 3 |  |  | | |
| IYCF | 53 | -0.41(-0.99,0.16) | 0.129 | 0.527 |
| PC3 | 53 | -0.03(-0.16,0.09) | 0.731 | 0.877 |
| PC3-by-IYCF^3^ | 53 | 0.16(-0.04,0.36) | 0.176 | 0.281 |
| PCoA Axis 4 |  |  | | |
| IYCF | 53 | -0.33(-0.90,0.23) | 0.216 | 0.649 |
| PC4 | 53 | 0.00(-0.14,0.14) | 0.9986 | 0.986 |
| PC4-by-IYCF^3^ | 53 | -0.13(-0.36,0.09) | 0.301 | 0.401 |

^1^Covariates include birthweight, infant sex, age at the 6mo visit, infant LAZ at the 6mo visit, infant diet diversity score at the 6mo visit, and mother-infant Lewis-null secretor phenotype discordance coded as both, none, infant only or mother only

^2^Adjusted for multiple hypothesis testing by the Benjamini-Hochberg method

^3^LAZ differences-in-differences

ΔLAZ, difference in length-for-age z-score; 95%CI, 95% confidence interval; IYCF, infant and you child feeding

**Table S9. Analysis of Variance for Distance Matrices (adonis2) using Bray-Curtis Dissimilarities to identify infant characteristics that significantly explain variation in microbiome composition using 354 fecal metagenomes collected from 1mo to 18mo from 172 infants.**

|  |  | **Un-adjusted Models** | |  | **Full Model** | |
| --- | --- | --- | --- | --- | --- | --- |
|  | **N/specimens^1^** | **% Variance Explained (R^2^ x 100)** | **p-value** | **N/specimens^1^** | **% Variance Explained (R^2^ x 100)** | **p-value** |
| Infant Age (mo) | 170/348 | 12.64% | 0.001 | 170/348 | 12.61% | 0.001 |
| Female Infant | 172/354 | 0.30% | 0.521 |  |  |  |
| EBF (3mo) | 87/226 | 0.46% | 0.483 |  |  |  |
| Season of Birth (Rainy vs Dry) | 172/354 | 0.33% | 0.472 |  |  |  |
| Minimum Infant Dietary Diversity Score Met | 158/330 | 0.30% | 0.521 |  |  |  |
| Food Insecurity | 169/349 | 0.34% | 0.460 |  |  |  |
| Mother-Infant FUT2 Concordance | 172/354 | 0.88% | 0.750 |  |  |  |
| Mother-Infant FUT3 Concordance | 172/354 | 1.02% | 0.476 |  |  |  |
| Mother-Infant FUT2-/FUT3+ Concordance | 172/354 | 0.68% | 0.741 |  |  |  |
| Mother-Infant FUT2+/FUT3+ Concordance | 172/354 | 0.73% | 0.651 |  |  |  |
| Mother-Infant FUT2+/FUT3- Concordance | 172/354 | 1.02% | 0.445 |  | 0.81% | 0.323 |

^1^Total unique infants / total number of specimens included in the analysis

**Table S10. Multivariable regression models^1^ to estimate modification of IYCF on LAZ at 18mo by infant gut microbiome species in 53 infants**

|  | **N** | **ΔLAZ (95%CI)** | **p-value** | **Adjusted p-value^2^** |
| --- | --- | --- | --- | --- |
| Bifidobacterium longum |  |  | | |
| IYCF | 53 | 0.35(-0.00,0.70) | 0.052 | 0.343 |
| B.longum | 53 | 0.44(0.12,0.76) | 0.007 | 0.067 |
| B.longum-by-IYCF^3^ | 53 | -0.86(-1.20,-0.52) | <0.001 | <0.001 |
| Bifidobacterium pseudocatenulatum |  |  | | |
| IYCF | 53 | 0.28(-0.13,0.69) | 0.026 | 0.343 |
| B.pseudocatenulatum | 53 | -0.55(-0.98,-0.12) | 0.012 | 0.077 |
| B.pseudocatenulatum-by-IYCF^3^ | 53 | 0.55(0.11,0.99) | 0.012 | 0.070 |
| Escherichia coli |  |  | | |
| IYCF | 53 | 0.03(-0.31,0.36) | 0.866 | 0.977 |
| E.coli | 53 | 0.07(-0.28,0.41) | 0.704 | 0.846 |
| E.coli-by-IYCF^3^ | 53 | 0.10(-0.25,0.45) | 0.571 | 0.792 |
| Dorea longicatena |  |  | | |
| IYCF | 53 | -0.00(-0.34,0.33) | 0.977 | 0.977 |
| D.longicatena | 53 | 0.23(-0.17,0.63) | 0.255 | 0.722 |
| D.longicatena-by-IYCF^3^ | 53 | -0.16(-0.70,0.38) | 0.572 | 0.792 |
| Dorea formicigenerans |  |  | | |
| IYCF | 53 | 0.27(-0.05,0.60) | 0.101 | 0.426 |
| D.formicigenerans | 53 | 0.02(-0.38,0.43) | 0.912 | 0.960 |
| D.formicigenerans-by-IYCF^3^ | 53 | 0.54(-0.35,1.43) | 0.232 | 0.608 |

^1^Covariates include birthweight, infant sex, age at the 6mo visit, infant LAZ at the 6mo visit, infant diet diversity score at the 6mo visit, and mother-infant Lewis-null secretor phenotype discordance coded as both, none, infant only or mother only

^2^Adjusted for multiple hypothesis testing by the Benjamini-Hochberg method

^3^LAZ differences-in-differences

ΔLAZ, difference in length-for-age z-score; 95%CI, 95% confidence interval; IYCF, infant and you child feeding

**Table S11. Multivariable mixed-effects zero-inflated beta regression to identify predictors of *Bifidobacterium longum* relative abundance**

|  |  | **Present vs Absent** | |  | **Relative Abundance if Present** | |
| --- | --- | --- | --- | --- | --- | --- |
|  |  |  | |  |  | |
|  | **n/N^1^** | **OR (95%CI)** | **p-value** | **N^2^** | **Rel. Ab. Ratio (95%CI)** | **p-value** |
| Infant Age (mos) | 179/225 | 1.02(0.97,1.07) | 0.517 | 179 | 0.91(0.90,0.92) | **<0.001** |
| Infant Sex |  |  |  |  |  |  |
| Male | 97/122 | Ref |  | 122 | Ref |  |
| Female | 82/103 | 0.97(0.49,1.91) | 0.927 | 103 | 0.91(0.82,1.01) | 0.080 |
| Ever EBF at 3mo |  |  |  |  |  |  |
| No | 35/39 | Ref |  | 39 | Ref |  |
| Yes | 144/186 | 2.24(0.73,6.85) | 0.159 | 186 | 1.16(0.99,1.37) | 0.066 |
| Mother-Infant FUT2+/FUT3- Phenotype |  |  |  |  |  |  |
| Both | 19/25 | Ref |  | 25 | Ref |  |
| None | 111/139 | 0.92(0.32,2.65) | 0.883 | 139 | 0.98(0.83,1.14) | 0.754 |
| Infant only | 19/25 | 1.01(0.27,3.84) | 0.987 | 25 | 0.93(0.75,1.14) | 0.471 |
| Mother only | 30/36 | 0.81(0.22,3.03) | 0.751 | 36 | 0.71(0.59,0.87) | **0.001** |
| Infant Dietary Diversity |  |  |  |  |  |  |
| Minimum Score Not Met | 87/116 | Ref |  | 116 | Ref |  |
| Minimum Score Met | 92/109 | 1.66(0.84,3.28) | 0.148 | 109 | 0.98(0.90,1.07) | 0.663 |

^1^Number of specimens with *Bifidobacterium longum* detected / Total number of specimens

^2^Number of specimens with *Bifidobacterium longum* detected

**Table S12. Multivariable logistic regression to identify predictors of *Bifidobacterium longum* strain cluster prevalence**

|  |  | ***B. infantis* cluster** | |  | ***B. longum longum* cluster** | |  | ***B. suis* cluster** | |
| --- | --- | --- | --- | --- | --- | --- | --- | --- | --- |
|  | **n/N^1^** | **OR(95%CI)** | **p-value** | **n/N^1^** | **OR(95%CI)** | **p-value** | **n/N^1^** | **OR(95%CI)** | **p-value** |
| Infant Age (mos) | 119/133 | 0.85(0.75,0.96) | **0.008** | 4/133 | 1.13(1.00,1.27) | **0.043** | 5/133 | 1.15(1.03,1.29) | **0.011** |
| Infant Sex |  |  |  |  |  |  |  |  |  |
| Male | 61/63 | Ref |  | 3/70 | Ref |  | 9/70 | Ref |  |
| Female | 58/70 | 8.35(3.17,21.97) | **<0.001** | 1/63 | 0.42(0.15,1.14) | 0.089 | 1/63 | 0.11(0.04,0.34) | **<0.001** |
| Ever EBF at 3mo |  |  |  |  |  |  |  |  |  |
| No | 25/28 | Ref |  | 1/28 | Ref |  | 2/28 | Ref |  |
| Yes | 94/105 | 1.09(0.29,4.09) | 0.895 | 3/105 | 0.79(0.27,2.34) | 0.667 | 8/105 | 0.93(0.23,3.70) | 0.92 |
| Mother-Infant FUT2+/FUT3- secretor Phenotype |  |  |  |  |  |  |  |  |  |
| Both | 15/16 | Ref |  | 0/16 | Ref |  | 1/16 | Ref |  |
| None | 68/79 | 0.67(0.19,2.28) | 0.518 | 4/79 | 1.92(0.48,7.71) | 0.356 | 7/79 | 0.82(0.23,2.95) | 0.756 |
| Only Infant | 14/14 | 4.66(1.05,20.71) | **0.043** | 0/14 | 1.02(0.25,4.12) | 0.978 | 0/14 | 0.21(0.05,0.90) | **0.036** |
| Only Mom | 22/24 | 0.77(0.09,6.75) | 0.812 | 0/24 | 0.84(0.20,3.49) | 0.814 | 2/24 | 1.19(0.15,9.32) | 0.871 |
| Infant Dietary Diversity |  |  |  |  |  |  |  |  |  |
| Minimum Score Not Met | 61/54 | Ref |  | 2/61 | Ref |  | 5/61 | Ref |  |
| Minimum Score Met | 65/72 | 1.39(0.43,4.50) | 0.582 | 2/72 | 0.84(0.31,2.30) | 0.737 | 5/72 | 0.72(0.18,2.90) | 0.647 |

^1^Number of specimens with a strain belonging to each cluster detected / Total number of specimens

**Supplemental Data File 1**

**Table S13. Overrepresentation of UniProt gene families and Metacyc pathways, which differ between strain clusters, by GO biological process, CAZyme or Transporter class or Metacyc pathway type.**
